# Leveraging the fundamentals of heat transfer and fluid mechanics in microscale geometries for automated next-generation sequencing library preparation

**DOI:** 10.1038/s41598-024-63014-x

**Published:** 2024-05-31

**Authors:** Olivia Ott, Sabrina Tolppi, Jennifer Figueroa-Cruz, Khaliun Myagmar, Khulan Unurbuyan, Anubhav Tripathi

**Affiliations:** Center for Biomedical Engineering, School of Engineering, Brown University, Providence, RI USA

**Keywords:** Biomedical engineering, Assay systems, Sequencing, Biological techniques, Biotechnology, Engineering

## Abstract

Next-generation sequencing (NGS) is emerging as a powerful tool for molecular diagnostics but remains limited by cumbersome and inefficient sample preparation. We present an innovative automated NGS library preparation system with a simplified mechanical design that exploits both macro- and microfluidic properties for optimizing heat transfer, reaction kinetics, mass transfer, fluid mechanics, adsorption–desorption rates, and molecular thermodynamics. Our approach introduces a unique two-cannula cylindrical capillary system connected to a programmable syringe pump and a Peltier heating element able to execute all steps with high efficiency. Automatic reagent movement, mixing, and magnetic bead-based washing with capillary-based thermal cycling (capillary-PCR) are completely integrated into a single platform. The manual 3-h library preparation process is reduced to less than 15 min of hands-on time via optimally pre-plated reagent plates, followed by less than 6 h of instrument run time during which no user interaction is required. We applied this method to two library preparation assays with different DNA fragmentation requirements (mechanical vs. enzymatic fragmentation), sufficiently limiting consumable use to one cartridge and one 384 well-plate per run. Our platform successfully prepared eight libraries in parallel, generating sequencing data for both human and *Escherichia coli* DNA libraries with negligible coverage bias compared to positive controls. All sequencing data from our libraries attained Phred (Q) scores > 30, mapping to reference genomes at 99% confidence. The method achieved final library concentrations and size distributions comparable with the conventional manual approach, demonstrating compatibility with downstream sequencing and subsequent data analysis. Our engineering design offers repeatability and consistency in the quality of sequence-able libraries, asserting the importance of mechanical design considerations that employ and optimize fundamental fluid mechanics and heat transfer properties. Furthermore in this work, we provide unique insights into the mechanisms of sample loss within NGS library preparation assays compared with automated adaptations and pinpoint areas in which the principles of thermodynamics, fluid mechanics, and heat transfer can improve future mechanical design iterations.

## Introduction

**Figure 1:**
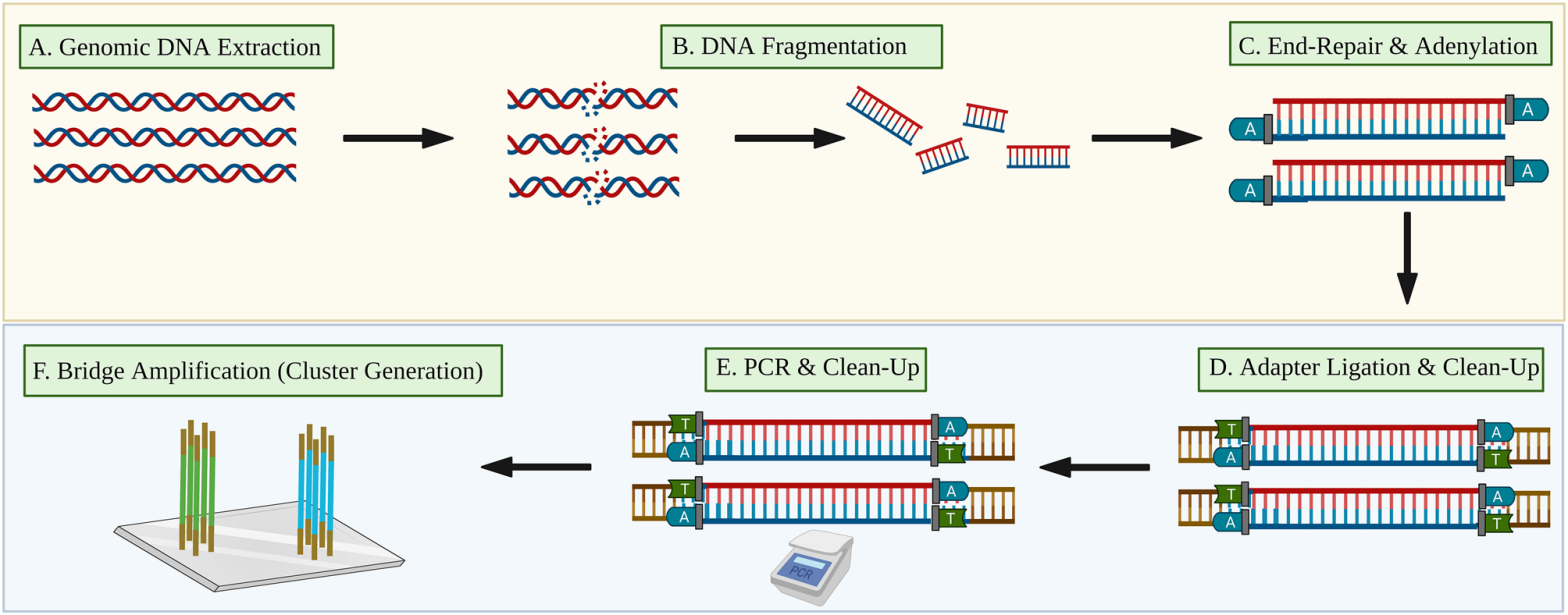
A typical NGS library preparation workflow. (**A**) Genomic DNA is extracted from blood or another sample matrix, (**B**) gDNA is mechanically, enzymatically, or chemically fragmented, (**C**) DNA fragments are blunted, adenylated, 5' phosphorylated, and (**D**) ligated to indexed adapters. Primer-dimers are removed in a SPRI bead purification step. (**E**) Adapter-ligated DNA fragments are PCR amplified and a second SPRI bead purification step removes fragments < 200 bp. (**F**) Final libraries undergo bridge amplification for cluster generation on the NGS flow cell^21^.

Next-generation sequencing (NGS) has transformed the biomedical landscape and ushered in a new age of discovery in genomics, significantly impacting disciplines such as cancer research^1–4^, rare disorder diagnosis^5–7^, and prenatal screening^8^. NGS is a markedly valuable tool for diagnostics and discovery because it generates millions of reads and is exceedingly sensitive to nucleic acid mutations^9–12^. Unfortunately, while innovations in sequencing technology have reduced requisite cost, time, and workload, sample (library) preparation has largely remained rudimentary and labor-intensive^13–16^. Even for applications requiring less data, such as microbial whole genome sequencing (WGS) or RNA-seq, the majority of the total sequencing cost can be attributed to library preparation, a critical upstream process that ultimately determines the success or failure of a sequencing run^17^. A typical NGS library preparation begins with the extraction of genomic DNA (gDNA)^18^ (Fig. 1A) followed by mechanical or enzymatic fragmentation (Fig. 1B). Sufficiently large yield from nucleic acid extraction is required to ensure high library complexity, while a fragmentation method that does not introduce bias is critical^19,20^. The dsDNA fragments must then be ligated to indexed adapters or primers that will eventually enable hybridization to the NGS flow cell for cluster generation (Fig. 1F). To achieve this, the ends of the fragments are blunted, adenylated, and 5' phosphorylated (Fig. 1C)^16^. A ligase enzyme is then able to attach the indexed adapters to the A-overhangs (Fig. 1D). This reaction generates unwanted by-products, known as adapter-dimers, when adapters are ligated to one another rather than to the DNA inserts. Adapter-dimers can be problematic since they will readily hybridize and amplify on the sequencing flow cell, taking the place of DNA inserts and providing useless sequencing data. A post-ligation purification step is thus typically performed using solid phase reversible immobilization (SPRI), a method that employs paramagnetic beads (MBs) to selectively bind larger DNA fragments^16^. Purification steps are sensitive and time-consuming, requiring large-volume washes and pipette mixing. Following purification, adapter-ligated DNA fragments are amplified via a polymerase chain reaction (PCR) to generate input library concentrations of > 5 ng/µL for the NGS flow cell (Fig. 1E). A post-PCR purification step is necessary to remove unwanted primer and adapter dimers, nucleotides, and any DNA fragments less than 200 bp in length^14,16^.

It is clear that NGS sample preparation requires many sequential steps using specialized laboratory equipment. Hence, NGS library preparation is expensive, cumbersome, and susceptible to contamination and pipettor error^22–25^. For small clinics and genomics laboratories, the use of NGS continues to hinge on the success of error-free manual library preparation done by an experienced scientist. Contamination remains the most persistent issue, but the risk can be mitigated by reducing human interaction with both reagents and samples via automation solutions.

Most automated NGS library prep methods focus on the implementation of robotic liquid handling for accurate pipetting of low and high-viscosity liquids^26^, integration of thermal cycling units, and inclusion of magnetic capabilities enabling bead-based purification and size selection. Liquid handlers often rely on the one-to-one adaptation of manual processes using robotic arms, demanding additional hardware, such as plate grippers, motion sensors, pipette tip collectors, firmware controllers, etc., thereby significantly increasing the cost of library preparation per sample. In addition to substantial initial investment costs, liquid handling workstations require expert programmers to create scripts for the purpose of adapting new workflows onto specific platforms and workarounds to combat reagent evaporation and tackle high-viscosity liquids. As a result, pipetting workstations are suitable only for high throughput applications that require massively parallel data collection.

Expensive, high-throughput robotic liquid handlers dominate research and clinical applications in core laboratories, however small laboratories often have neither the funds nor the sample demand to warrant a high-throughput automation solution. Small laboratories are consequently often restricted to labor-intensive manual protocols that introduce many opportunities for sample contimination and manual pipetting errors due to various factors. Microfluidic approaches are one group of solutions often more suitable for low-throughput and single-use library preparation applications^14,16,24,27–29^. Single-use microfluidic chips harbor risks for the environment, producing consequential waste and requiring safe disposal if toxic or dangerous chemicals are incorporated into chip design. Select microfluidic approaches, such as the nanoliter-scale column chromatography method described by Tan et. al., have scaled successfully to be high-throughput and commercially viable^30^. Tan et. al. utilized a novel architecture, with a motif of two repeating fluidic circuits that employ a combination of commercially available reagents to achieve library preparation with minimal manual intervention. Still, full automation at the nanoscale for hundreds of parallel reactions demands low-tolerance chip manufacturing and integration with a high-throughput liquid handler, resulting in high investment costs for its associated equipment. Consequently, expensive robotic liquid handlers dominate research and clinical applications in core laboratories, and small laboratories suffer from the bottleneck of labor-intensive manual protocols. Automation innovation for small laboratories is necessary to accelerate NGS-based research, eliminate sources of human error, and reduce hands-on time^13–16^. Our previous work started with the innovative concept of integrating liquid mixing steps with thermodynamically driven steps such as ligation and amplification via a single capillary tube heating system^13–16^. This work utilized magnetic bead motion through a microchannel for purification step, as needed by steps C and D in Fig. 1. Schneider et al. achieved a high level of integration with minimal hands-on time, yet only one sample could be prepared at a time, with some concerns of microfluidics chip cost and manufacturability. Additionally, scalability of microfluidics chips for many library preparation chemistries, each requiring different amounts of bead solution, is sometimes operationally challenging. Hence, the initial proof-of-concept platform required further innovation for it to be globally adopted by researchers and clinicians with reduced complexity and cost, and a high degree of robustness and ease-of-use.

**Figure 2:**
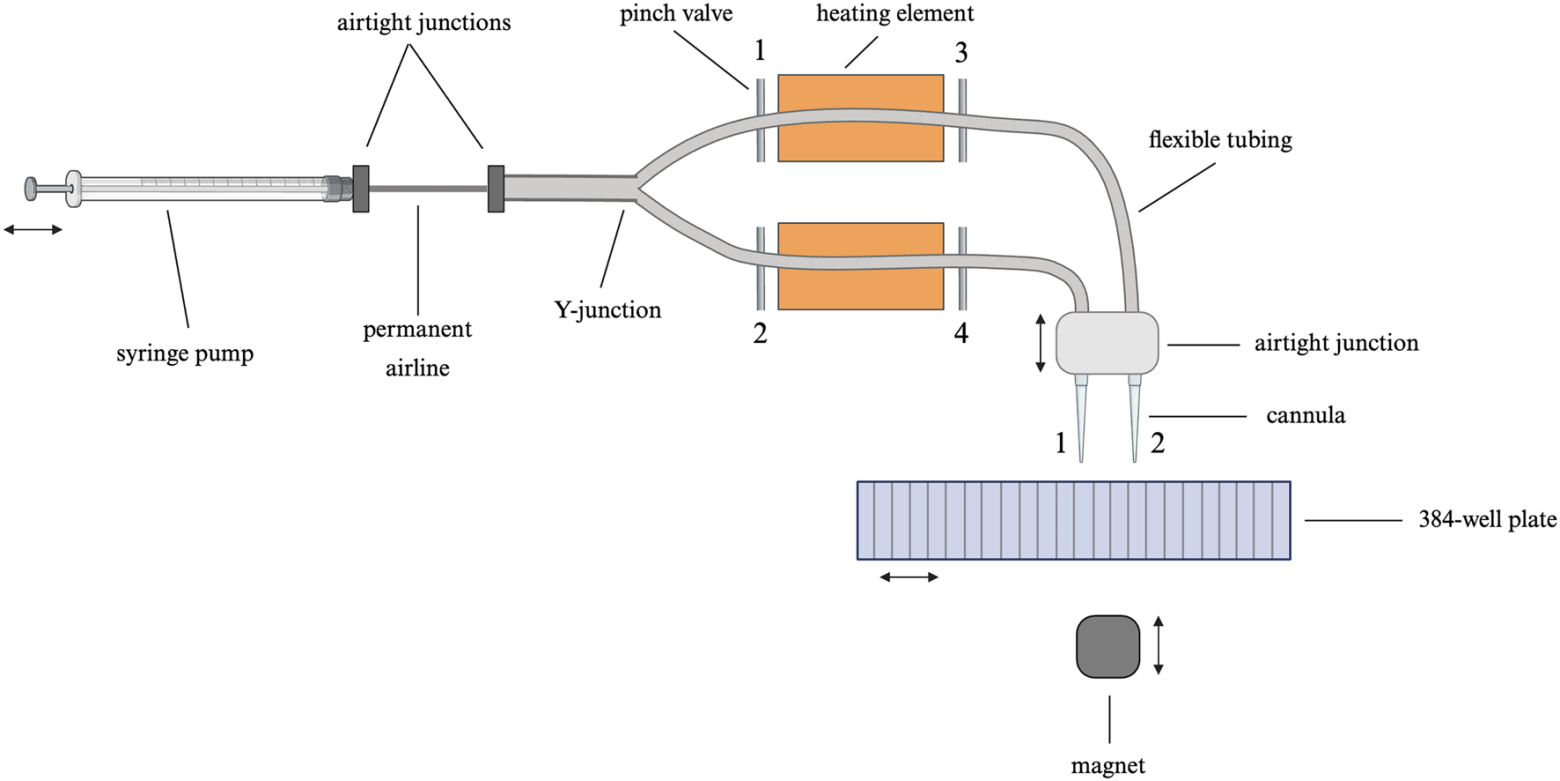
Schematic of our platform hardware, simplified to illustrate components for preparation of one sample. The air pressure syringe pumps are placed on an x-axis motion stage, the cannulas are placed on a z-axis motion stage, the 384-well reagent plate is placed on an x-stage, and the magnet is put on a z-axis motion stage.

In this work, we present an innovative automated NGS library preparation system with a simplified mechanical design that exploits both macro- and microfluidic properties. As shown in Fig. 2, an air-displacement syringe pump is connected to two cannulas via permanent airlines then disposable flexible tubing that passes through pinch valves and a heating element. Cannula 1 goes into the desired wells of the 384-well reagent plate to aspirate, dispense, and mix reagents, then transport necessary mixtures to the heating element (as desired in steps B, D and E in Fig. 1) with pinch valves 2 & 4 in the closed position. Similarly, cannula 2 operates with pinch valves 1 & 3 in the closed position. A magnet, which is located beneath the 384-well plate, is used for magnetic bead-based purification of nucleic acids as needed by steps C and E in Fig. 1. The pinch valves are used to pressurize the air surrounding liquid plugs in the heating section undergoing ligation or PCR. Our modular platform integrates fluid mechanics, heat transfer, molecular thermodynamics, mass transport, and biochemical reaction kinetics.

Our refined design allows for consistent operation of eight library preparations in parallel, while maintaining low manufacturing and operational costs without any need of microfluidic chip-based bead cleaning. The platform’s use of a 384 deep-well plate for all liquid handling offers a centralized, efficient, and adaptable workflow. As a result, the manual 3-h library preparation process is reduced to less than 15 min of hands-on time from the start of the procedure, followed by less than 6 h of instrument run (walk-away) time. This approach allows us to successfully prepare eight libraries in parallel in under 6 h and obtain high-quality sequencing data for both human and *E. coli* DNA with negligible coverage bias. We applied this method to two library preparation assays with different DNA fragmentation methods (one mechanical and one enzymatic), simplifying consumable use to one cartridge (16 cannula or ‘pipette’ tips) and one 384-well plate per eight libraries (post-automated and/or manual pre-plating of reagents). Although in this work we demonstrate NGS library preparation assay automation, our innovative platform could be easily programmed to perform nucleic acid extraction protocols, various end-point PCR assays, and protein detection assays utilizing the same reagent plate and cartridge consumables. Our platform combines the reliability of large pipetting workstations with the low cost and enclosed environment of microfluidic solutions, while striking a balance between integration and versatility.

## Results and discussion

### Optimization of mechanical design

**Figure 3:**
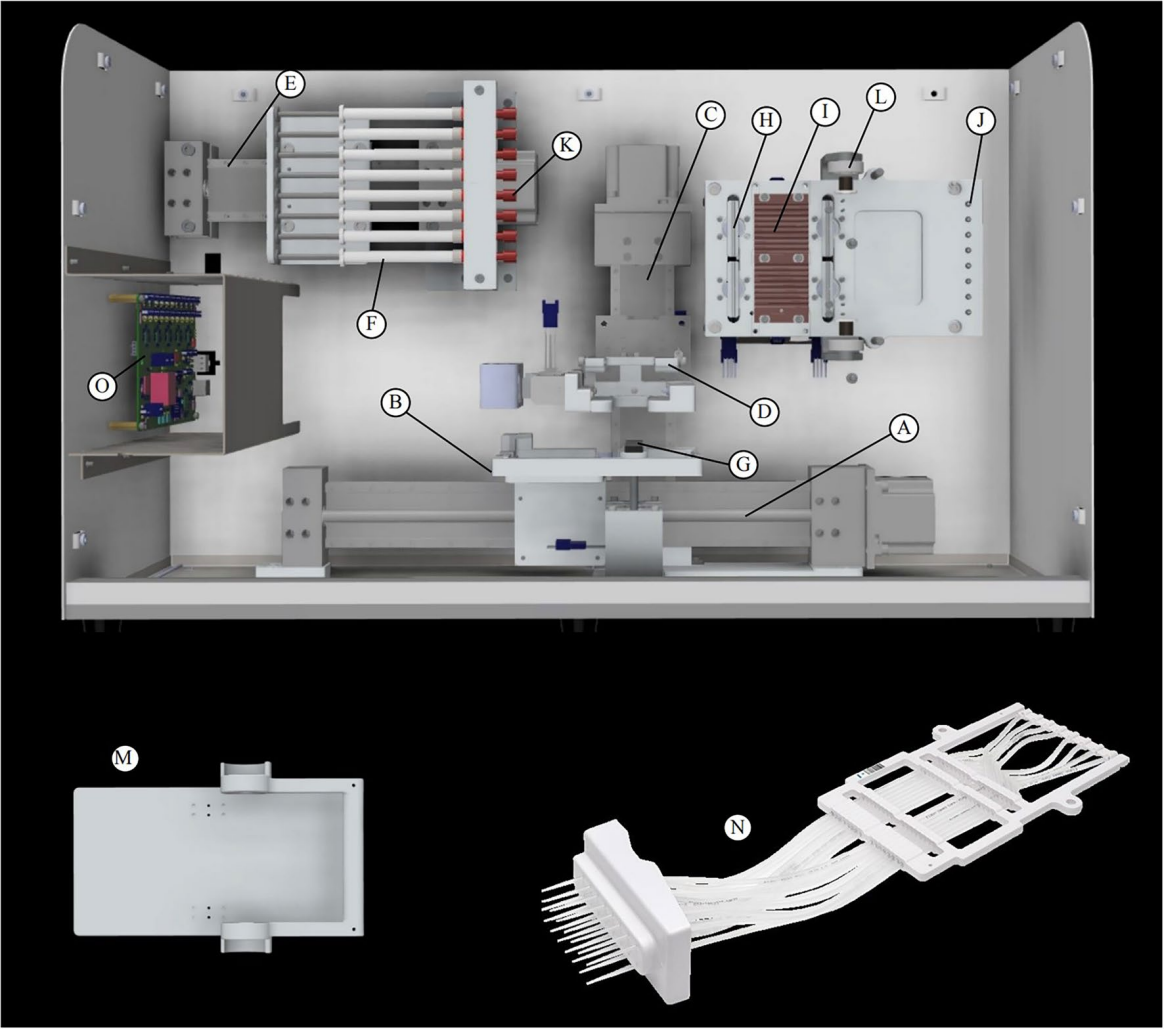
Schematic of our platform. (**A**) X-stage for reagent plate motion, (**B**) 384 deep-well plate holder, (**C**) Z-stage for cannula motion, (**D**) Cartridge cannula spring-loaded clamp, (**E**) X-axis for pump syringe motion, (**F**) Syringes, (**G**) Magnet in raised position for bead-based purification, (**H**) Pinch valve, (**I**) Copper heating plate, (**J**) O-rings for cartridge-airline interface, (**K**) Airline connection point for plastic tubing (not pictured) to extend to (**J**), (**L**) Anchoring point for (**M**) insulated PCR door, (**N**) Disposable cartridge with cannula tips forms an airtight seal with (**J**) and is secured to the device via (**M,D**). (**O**) Printed circuit board for platform.

The principal innovation of our method is the simplified design layout of the platform (Figs. 2, 3) based on the fundamental needs and constraints of fluidics, reactions, adsorption–desorption, and mixing. All NGS library preparation workflows (Fig. 1) require several reagents and buffers at working volumes between 2 and 200 µL. To accommodate these working volumeswe found that it is optimal to use a 384 deep-well plate with the well dimensions: 3.48 mm(*w*) X 3.48 mm(*w*) X 19.3 mm(*L*). Narrow dimensions of wells offer faster diffusional ($\tau_{d} \sim w^{2} /2D$) and convective ($\tau_{c} \sim w^{2} L/Q$) mixing. Here, *D* is the molecular diffusivity, and *Q* is the flow rate of liquid dispensing. A deep-well plate also offers adequate space to arrange and store reagents wells to avoid any carry-over contamination. Deep wells, in addition to a pierceable plate seal, help to prevent aerosol contamination of nearby reagent wells during liquid handling. Aerosol contamination of the airlines is prevented by using sufficiently long segments of cartridge tubing, as well as slow pump speeds to maintain volumetric accuracy of pipetting. Our design strategy allows users to use pre-plated reagents for eight samples (Rows A, C, E, G, I, K, M, O) in the 384-well plate stored at -20 °C. The platform enables movement of the 384-well plate in only a single dimension (x-stage for left–right motion) (Fig. 3A,B). A separate z-stage (for up-down motion) (Fig. 3C,D) holds two cannulas (Fig. 3N) able to move together perpendicular to the x-stage for liquid transfers from any of 24 wells in a single row of the 384-well plate. The two cannulas are connected via polyethylene capillary tubing (Fig. 3N) which pass through a thermal heating plate (Fig. 3I) and a set of two pinch valves (Fig. 3H,I). The pinch valves are used to select one of two cannulas by pinching the undesired cannula against the PCR door, allowing for use of a single syringe pump to control a bifurcated airflow. Our platform offers the option of two separate cannulas (Fig. 3N) for various reactions in the library preparation workflow (Fig. 1). Our device uses a grooved copper plate that accomodades cartridge polyethylene tubing to maximize the thermal heating efficiency (Fig. 3I). Roughly one-third of the tubing directly contacts the copper surface and is held securely in place by an insulated detachable door (Fig. 3M). Lastly, our device uses a permanent magnet that can be moved in proximity to the base of the 384-well plate for bead-based purification of DNA. The magnet is mounted to a separate z- stage for up-down motion (Fig. 3G). To summarize, the psychology behind the design optimization was two-fold: (a) to keep the hardware simple, robust, and low-cost; (b) to use optimal fluidic and reaction time scales for integrating various steps needed in any library preparation workflow (Fig. 1).

### Fundamentals of assay design and optimization of variables

**Figure 4:**
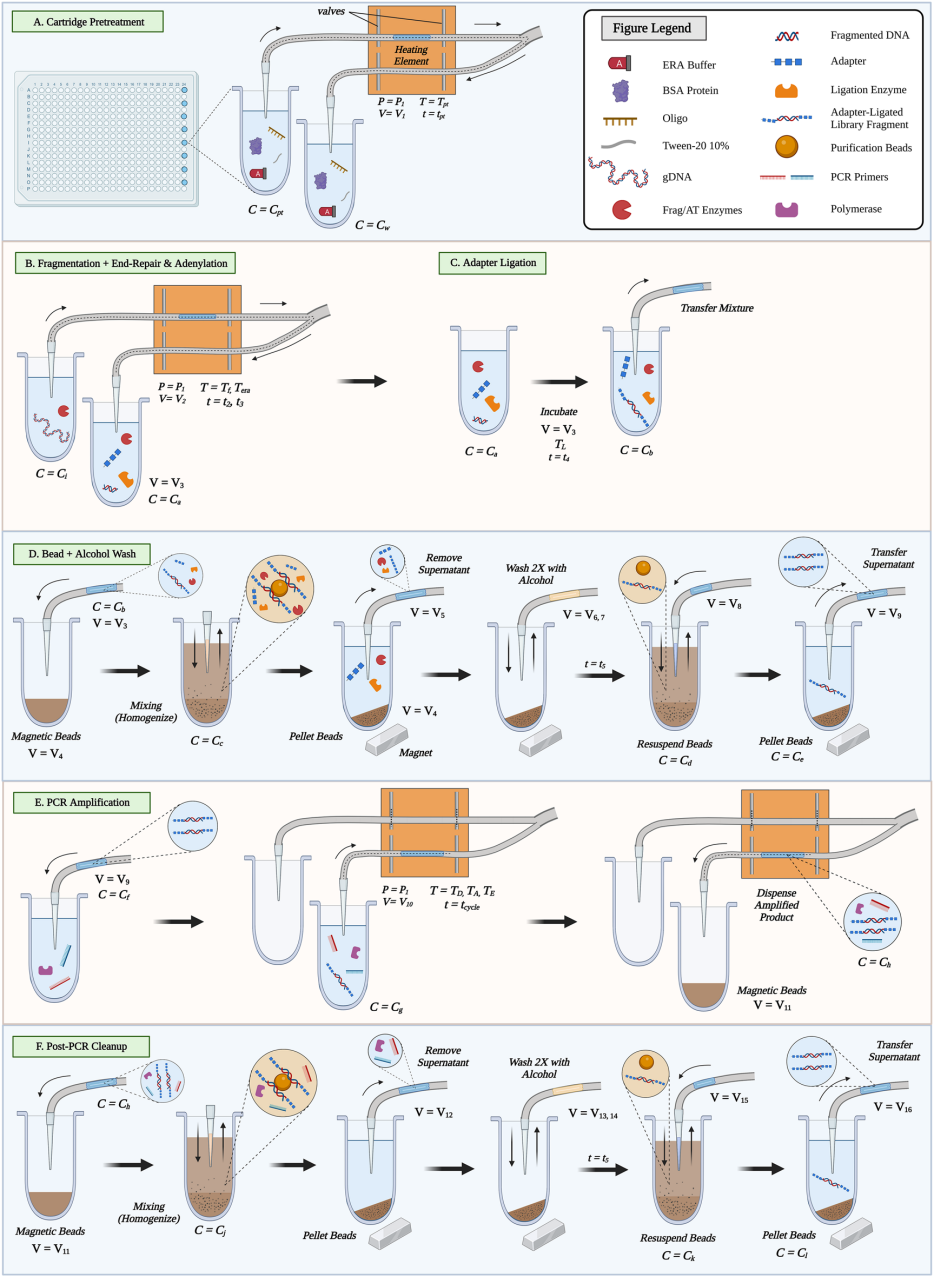
Details of our optimized method. Both the mechanical and enzymatic fragmentation assays follow the protocol depicted below, differing in select incubation times and reaction volumes (See S. Table 1). (**A**) Surface passivation (Pretreatment) of the cartridge tubing. (**B**) Fragmentation, end repair, and adenylation reaction at the heating element, (**C**) adapter ligation reaction, (**D**) post-ligation SPRI bead purification, (**E**) Capillary PCR amplification, (**F**) post-PCR SPRI bead purification. Variables T = temperature, t = time, P = pressure, V = volume, and C = concentration. Note that fragmentation only occurs on-platform for the enzymatic fragmentation assay. Cannula tip positions relative to the well are not exact, as there are multiple z-axis positions per step^21^.

**Figure 5:**
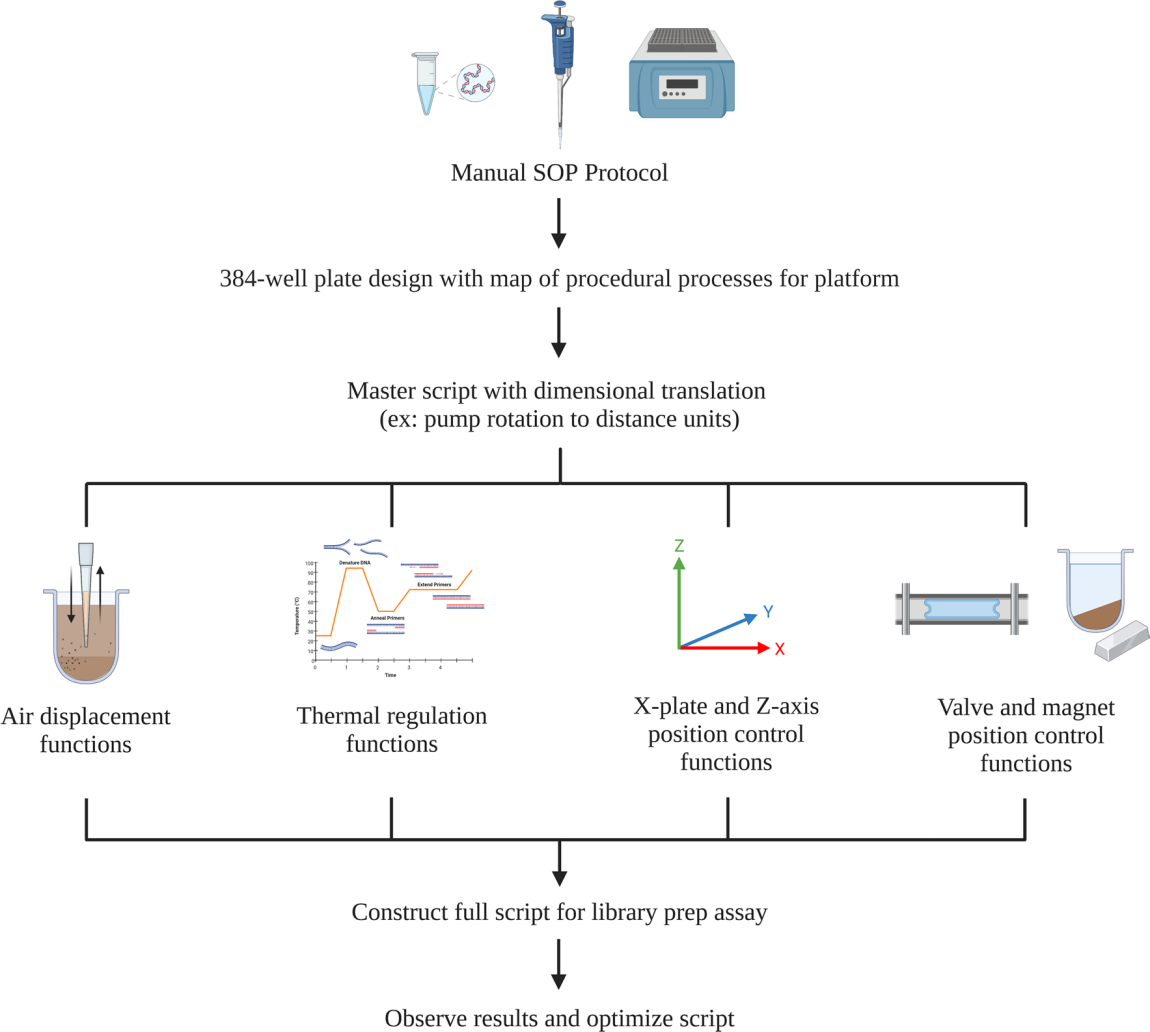
Scripting workflow. Standard operating procedure (SOP) for script and assay development^21^.

Our unique approach amalgamates multiple microfluidic and macrofluidic steps into one cohesive and comprehensive workflow. Unlike most liquid handling platforms, our design requires only two cannula tips per sample and corresponding capillary tubes to perform all liquid transfers and mixing functions for the entire library preparation assay. In development of the platform, we found that reuse of tubing following fragmentation or end repair and adenylation (ERA) reactions (Fig. 4B) negatively impacted subsequent steps, while adsorbed reagents from wash and PCR steps (Fig. 4C–F) were less likely to impair assay performance. As a result, one cannula is used for fragmentation or ERA reactions, while the other is reserved for all other steps (Fig. 4A,B,E). Our results below demonstrate that library preparation assays are surprisingly tolerant of surface reuse, allowing for design simplification as long as individual sample lanes do not contaminate each other. For a high-throughput liquid handler, similar surface reuse may be possible, though we anticipate that a full study of assay performance and cross-contamination would need to be conducted to ensure that there are no unintended consequences. We have optimized the use of the capillary tubes based on adsorption–desorption characteristics and by incorporating a cartridge surface passivation step that must be completed for every new run. Experiments were performed to optimize pre-treatment (PT) conditions using mixture components, fluid slug volume, slug routing path, temperature, and incubation time as variables. The pretreatment is comprised of water, Tween-20, ssDNA oligo, BSA protein, and ERA buffer (full mixture specified in S. Table 1A). Under optimal conditions, the PT reaction is mixed inside the 384-well plate, via up and down fluid motion in the cannula tips, before the entire reaction volume (V_1_ = 50 µL) is transported inside the capillaries to the heating element, undergoes heating for t_PT_ = 10 min at T_PT_ = 75 °C, and is passed through the Y-junction of the cartridge and discarded (Fig. 4A). This step successfully prevents the adsorption of enzymes and DNA to the polymeric surface of the cartridge tubing. Without the optimized PT, final library concentrations for the mechanical fragmentation assay average 11.42 ± 3.66 ng/µL compared to an average of 33.12 ± 6.54 ng/µL with the PT. In addition to low yield, these samples have tall adapter dimer peaks (S. Figure 5), highlighting the importance of PT for generating high quality libraries.

We optimized the fragmentation and ERA reaction volumes to be V_2_ = 50 µL for both assays (Fig. 4B). These reactions contain input DNA, buffer, and the appropriate fragmentation or ERA enzymes. Fluid mixing in this step is performed in the 384-well plate using repeated pipetting up and down at moderately slow speeds (S. Table 1)(see Results Section III for further discussion of mixing techniques and pump speeds). The fluid slug is transported in the capillaries to the heating element to achieve optimum enzyme activity at the desired temperature (room temperature incubation during the PT step then t = 30 min at T_era_ = 65 °C for mechanical fragmentation, and t_2_ = 15 min at T_f_ = 37 °C, t_3_ = 30 min at T_era_ = 65 °C for enzymatic fragmentation, Fig. 4B). Note that the fluid slug is constrained by pinch valves (Fig. 3H) and pressurized to applied pressure P_1_ = 1.90 atm during heating to eliminate any significant evaporation and further optimize heat transfer. Thereafter, the fluid slug is transported to a specified well in the plate containing adapters and ligase (Fig. 4C). The ligation reaction is mixed with a custom mixing technique (see Results Section III) and incubated at room temperature (T_L_ = 25 °C) for time t_4_ = 15 min. The adapter-ligated DNA sequences must then be purified to eliminate any non-ligated adapters and leftover enzymes. In this step, the liquid containing the adapter-ligated DNA fragments is transferred to a well containing SPRI beads (V_4_ = 100 µL for mechanical fragmentation, V_4_ = 60 µL for enzymatic fragmentation) (Fig. 4D). Several custom mixing techniques form the basis of the SPRI bead-binding and DNA elution reactions, with gentle alcohol washes in between. An optimized NGS library prep wash step is expected to remove any residual alcohol through bead drying and thorough removal of supernatant. The eluate of the wash step is mixed with PCR master mix and primers, then transported in the capillaries to the heating element to undergo thermal cycling for amplification (V_9_, Fig. 4E). The fluid slug is again pressurized to optimize heat transfer, and after performing the PCR cycle for a programmed amount of time (specified in S. Table 1E), the slug containing sample DNA is dispensed back into the plate for the final purification step. Post-PCR purification follows the same principles as post-ligation purification, with the goal of removing adapter and primer dimers, as well as remaining PCR reagents and small adapter-ligated DNA fragments < 200 bp (Fig. 4F). The final supernatant removal in this step containing the desired DNA library is dispensed as the final product in a specified plate column, ready for quantification and quality control (V_16_ = 30 µL for the mechanical fragmentation assay and V_16_ = 20 µL for the enzymatic fragmentation assay).

### Analysis of fluid mechanics and platform robotic control

When referring to positive controls, the amount of liquid lost during manual pipetting is assumed to be insignificant. Hence, our design is required to offer automated but reliable liquid transfers. However, automated liquid transfers typically result in inefficiencies at low volumes. Instead of adopting the expensive and very sophisticated pump assembly used by conventional liquid handlers, our design relies on a simple air-pressure syringe pump. The pump motor utilizes two instructions from the assay script (a) set position and (b) set velocity. The final position determined the pressure and speed was selected based on viscosity of the liquid. Our analysis of volumetric accuracy and %CV (S. Figure 2) suggests that only volumes less than 5µL resulted in a %CV higher than 10%. The error present is attributed to the limited accuracy and precision of the linear-rail motor driving syringe movement at small fractions of a shaft rotation. To mitigate this limitation, our platform is programmed to always transfer volumes greater than 5 µL, while the 384-well plate layout is designed so that low-volume reagent wells become liquid transfer destinations rather than liquid transfer sources. This method takes advantage of the pre-plating process for handling of critical low-volume reagents such as adapters, primers, and enzymes, avoiding on-platform volumetric loss. Assay development begins with manually performed variations of the SOP protocol, followed by reaction fine-tuning to adapt variables such as reagent volumes, reaction temperatures, and incubation times to our platform (Fig. 5). Once these parameters have been determined, a plate layout may be implemented, where assay reagents are arranged into columns of the 384-well plate (S. Figure 1). A master script, using Python as a programming language, establishes functions for air displacement, thermal regulation, X-plate control, Z-axis control, valve control, and magnet control that supplementary local scripts may call on for development of each individual assay (Fig. 5).

A preliminary assay script is written with the proposed plate layout, then liquid testing with dyed water in place of reagents is performed to identify potential mistakes, failures, or further points of iteration. The local script can be partitioned to perform liquid tests on individual steps of the assay, enabling the optimization of each step and function as individual segments. The script is then incrementally and experimentally adjusted until an optimized, robust, and automated assay is achieved.

Assay scripting is a highly informed process, with script writers aiming to control fluidic properties of the platform through refined motor movement. Relative reagent viscosity, estimated by manual pipetting, is used as a benchmark to select a pump speed for liquid transfers and mixing techniques. Operational pump speeds range from 1.67 µL/s (used only for liquid transfers of extremely viscous liquids) to 76.78 µL/s (used only for vigorous bead resuspension). Any newly implemented mixing or liquid transfer techniques must be assessed visually to ensure mixture homogeneity and precise aspiration and dispensation. High viscosity liquids must be aspirated and dispensed slowly, as they are prone to slow movement through cannula tips and cartridge tubing. Attempting high pump speeds with viscous liquids can result in volumetric inaccuracy or segmentation of fluid slugs. Moreover, techniques must avoid the introduction of bubbles in reaction mixtures, an indication of inappropriate pump speed or cannula tip positioning.

An example of these principles in practice is the custom ligation mixing technique. The ligation mixture of ligase enzyme, buffer, and adapters is highly viscous, and mixing must be done thoroughly yet carefully to prevent excessive adapter-dimer formation. Our mixing technique slowly aspirates a small volume of ligase enzyme/buffer, then a small volume of ERA/fragmentation mix combined with adapter, then repeats this process, alternating between the two mixtures. This technique increases surface area between the two mixtures, aiding diffusion while avoiding stringent mixing that may generate bubbles or adapter-dimers.

Bead-based purification steps have been a point of extensive script development, as these steps rely on liquid transfers orchestrated between many reagent wells (Fig. 4D,F) and custom mixing techniques that require precise movement of cannula tips. For bead peletting, the magnet is positioned to the side of the well containing the bead pellet. Cannula tips are always located in the center of the well, thus an offset bead pellet allows for aspiration of supernatant from the bottom of the well without aspiration of SPRI beads (and subsequent loss of yield). Dispensation of alcohol onto bead pellets is performed at medium–low pump speeds (S. Table 1) to avoid disturbing the pellet. For binding and elution mixing techniques, pump speeds alternate between fast (vigorous) and slow (more gentle) motion (S. Table 1) to vary flow of SPRI beads throughout DNA-reagent mixtures. Resuspension of bead pellets requires high speed dispensation (S. Table 1) of elution buffer onto the pellet with cannula tips positioned deep in the well, followed by several rounds of up and down mixing. Mixing techniques use mixture volume as a script function parameter to ensure that the cannula tips are never positioned above the fluid surface while aspirating, preventing bubble formation.

In order to perform assay steps requiring heating, an elaborate pressurization and depressurization routine is employed. Scripts instruct the fluid slug to approach the heating element, then close the left valve (Fig. 3H). The pump is then instructed to push air against the fluid slug (now immobilized by the left valve), reaching 1.90 atm. Finally, the right valve closes to encapsulate the pressurized, centered fluid slug for heating. Depressurization occurs with incremental movement of individual valves to prevent sudden movement of the fluid slug.

### Analysis of capillary heat transfer, pressurization, and diffusion

**Figure 6:**
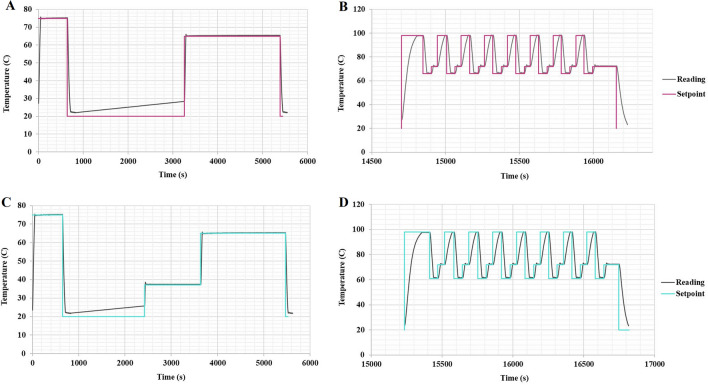
Comparison of thermocouple temperature readings and scripted setpoint temperatures vs. time for the duration of the mechanical fragmentation assay (**A**,**B**) and the enzymatic fragmentation assay (**C**,**D**). (**A**) Temperature vs. time for PT and ERA, and (**B**) temperature vs. time for PCR amplification. (**C**) Temperature vs. time for PT, fragmentation, and ERA, and (**D**) temperature vs. time for PCR amplification. Note that the temperature drift (**A**,**C**) when the setpoint is 20 °C is due to residual heat transferring to the block containing the thermocouple. Reagents are in the 384-well plate during this incubation time, so there is no negative effect on assay performance.

All reactions requiring heating are performed inside the cartridge capillaries in the form of cylindrical fluid slugs. For example, during enzymatic fragmentation, the 50 µL reaction mix is transported to the heating element at a specified pump velocity and subject to applied air pressure and temperature cycling: t_2_ = 15 min at T_f_ = 37 °C and t_3_ = 30 min at T_era_ = 65 °C, followed by a 4 °C hold (Fig. 4B). The maximum conductive heat flow rate through the cylindrical tube wall ($$Q)$$ during the reaction is estimated

1 $$Q = 2\pi krL\left( {\frac{\Delta T}{{\Delta r}}} \right)\sim {7}.{8}\;{\text{W}}$$

when the thermal conductivity^31^ of the polyethylene tube wall is $k\sim 0.33 \;\frac{{\text{W}}}{{{\text{m}}\;^\circ {\text{C}}}}$, the thickness of the tube wall is $\Delta r \sim 0.8\;{\text{mm}}$, the maximum temperature difference between the outer and inner surfaces (the conductive resistance) of the capillary is $\Delta T\sim 73$ °C, the tube radius is $r\sim 1.6\;{\text{mm}},$ and length of the slug is $L \sim 24.9\;{\text{mm}}.$ Similarly, the maximum conductive^32^ heat flow rate inside the fluid slug itself is $Q\sim 2\pi krL\left( {\frac{\Delta T}{{\Delta r}}} \right)$ ~ 6.8 W, where $r\sim 0.8\;{\text{mm}}$ and $k\sim 0.598\; \frac{{\text{W}}}{{{\text{m}}\;^\circ {\text{C}}}}$. Hence, the heat flow rates are approximately matched to achieve a rapid steady state between the tube and fluid slugs. The cylindrical shape of the fluid slug itself accelerates heat transfer

2 $$t_{d} \sim r^{2} /{2}\alpha \sim 2.2\;{\text{s}}$$

owing to microscale diffusional length. Here, α $= 0.15\; \frac{{{\text{mm}}^{2} }}{{\text{s}}}$ is the thermal diffusivity^32^ of the liquid, $t_{d}$ is the thermal diffusion time, and $r = 0.8\;{\text{mm}}$ is the radius of the fluid slug. Furthermore, the thermal capacitance of the fluid slug ~ $\rho \pi r^{2} L\left( {4.18} \right)\Delta T\sim 3.7 J$ is small compared to the conductive heat flow $Q\sim$ 6.8 W. Hence, the time scale for heating the plug is ~ $3.7/6.8 = 0.54\;{\text{s}}$, which is faster the timescale of resistive heat transfer. We purposely designed the Peltier heating element with a thermocouple embedded in a segment of cartridge tubing to mimic the thermal environment of the sample. Successful recreation of the sample environment allows our system to gather real-time, non-adjusted data of the reaction mixtures, which are slow to respond compared to the copper heating element. Our system logs temperature in real-time and compares measured temperatures against pre-scripted setpoints for reactions that require heating. A PID feedback mechanism ensures that the temperature of the fluid slug reaches the setpoint. The data, collected and stored in a text file, enables monitoring of system functionality and constant improvement of the device. Figure 6 shows the thermocouple temperature data compared against the scripted temperature setpoints for PT, fragmentation, ERA, and PCR during the mechanical fragmentation assay (Fig. 6A,B) and the enzymatic fragmentation assay (Fig. 6C,D). Total time for 8 cycles of PCR amplification is approximately 25 min (Fig. 6B,D), compared to 22 min on a standard thermal cycler. While our platform does have slightly slower ramp rates than a standard thermal cycler, we have not observed any significant primer/adapter dimer formation or negative impacts on amplification efficiency because of this slight difference. Statistical analysis following each run allows us to observe thermal profiles, calculate ramp rates of the heating element, and target specific modes of failure when developing new library preparation assays.

The pressurization of capillary slugs between two pinching valves expands the capillary polymer tube between the copper heating plate on one side and thermal insulation on the other. It improves the thermal contact area and increases the heat transfer from the copper to the liquid slug as described above. Although the water vapor permeability through polymeric tubing increases at higher temperatures^33^, the water loss in the system is minimal due to the lack of gradient water vapor concentration across the tube wall. Since the tubing is compressed between non-diffusive surfaces, the vapor quickly saturates the pores in the polymer tubing. A systematic study for quantifying evaporative loss at varying pressures is beyond the scope of this work, however, we did confirm that any volume loss was comparable to positive controls following heating steps. Note that pressurization likely prevents any bubble formation thatmay occur within fluid slugs, moreover, our data does not show any impact in amplification efficiency compared to positive controls. Given the importance of maintaining pressure for achieving optimal heat transfer, we conduct frequent analysis of system performance using a pressure sensor-equipped cartridge. This evaluation involves moving the syringe assembly to various positions to simulate high-pressure and vacuum conditions. Pressure is plotted over time (S. Figure 3) to acquire leak rates of the system and isolate specific airlines, syringes, or valves that may cause abnormal pressure readings.

Our thermal system design offered desired temperature profiles (as shown in Fig. 6) for PCR and ligation reactions as performed in a conventional manual method. PCR-induced bias, a common mode of failure in traditional automated platforms, is often a result of poor temperature and ramp rate regulation^34^; our sequencing results show no noticeable bias offered by thermal design and feedback system.

### Library preparation efficiency

Library preparation efficiency is an important metric to quantify the success of our automated method compared to manually prepared positive controls (note that all positive controls discussed are prepared manually). Efficient library preparation has implications for sequencing success, clinical relevance, and conservation of resources such as cost and requisite input sample volume.

Our approach was to first prepare four on-platform plates, each with a separate set of manual positive controls: mechanical fragmentation assay with (1) human and (2) *E. coli* DNA input, and enzymatic fragmentation assay with (3) human and (4) *E. coli* DNA input. These four plates are analyzed below for final library concentration, library fragment distribution, as well as numerous sequencing metrics. After successful completion of these four plates, we gathered data across 7 on-platform plates for the mechanical fragmentation assay and compared intra-run and inter-run consistency of final library concentration to positive controls to ensure robustness and repeatability of the instrument.

#### Controls

**Figure 7:**
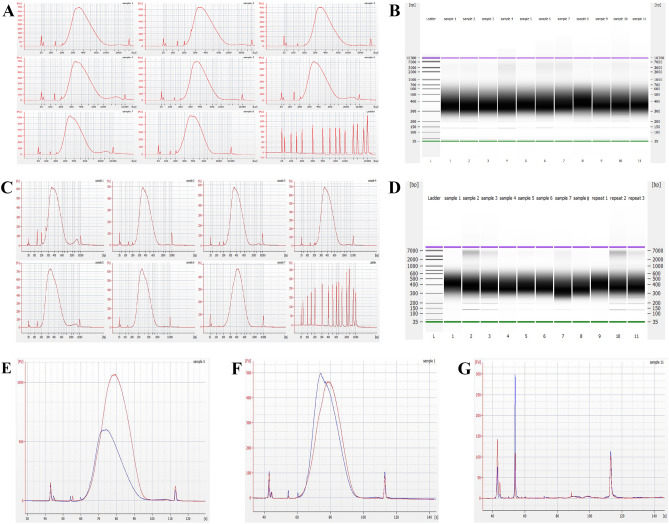
Mechanical fragmentation final library Quality Control. (**A**) Electropherograms of on-platform (Samples 1–6) and manual positive control (Samples 7–8) human DNA libraries. The bottom right panel shows a DNA ladder for size comparison. (**B**) Gel image demonstrating fragment size distributions of the human DNA libraries shown in (**A**). (**C**) Electropherograms of on-platform (Samples 1–6) and positive control (Sample 7) *E. coli* DNA libraries. The bottom right panel shows a DNA ladder for size comparison. (**D**) Gel image demonstrating fragment size distributions of the *E. coli *DNA libraries shown in C. (**E**) Human DNA positive control (red) and on-platform (blue) library overlay for yield and size distribution comparison. (**F**) *E. coli *DNA positive control (red) and on-platform (blue) library electropherogram overlay for yield and size distribution comparison. (**G**) Manual (red) and on-platform (blue) negative control electropherogram overlay. All electropherograms consist of lower (first) and upper marker (last) peaks. Adapter-dimer peaks present at ~ 130 bp (or ~ 55 s). Other tiny peaks are either pertaining to primers or noise.

Manual positive controls consisting of 20 ng of sheared DNA (*E. coli* and human, average 300 bp) were constructed for genomic libraries intended for whole genome sequencing (WGS), to confirm the validity of our on-platform mechanical fragmentation assay. Successful positive controls indicate that input DNA and reagents are within the concentrations and shelf life specified for the kit. Electropherograms and gel images illustrate the final library fragment size distribution for each sample and positive control (Fig. 7A–D), with positive control average fragment size ~ 400 bp for human libraries and ~ 420 bp for *E. coli* libraries. Positive control average yield and percent adapter-dimer were 912.22 $\pm 168.13 {\text{ng}}$ and 0.15 $\pm$ 0.00040% for human DNA and 445.69 ng and 0.10% for *E. coli* DNA, respectively (S.Table 3A). This data is consistent with our passing criteria: yield ≥ 250 ng total, concentration ≥ 5 ng/µL, adapter-dimer ≤ 5%, and fragment size distribution between 200 and 1000 bp, with an average close to 400 bp. A manual negative control was performed without input DNA, to evaluate any potential reagent contamination, while another on-platform negative control without DNA input assessed potential system contamination (Fig. 7G). The negative control electropherogram overlay shows a single peak between the upper and lower markers around 150 bp corresponding to adapter-dimer, consistent with the lack of input. No erroneous peaks were observed, indicating that the reagents and system are contamination-free.

**Figure 8:**
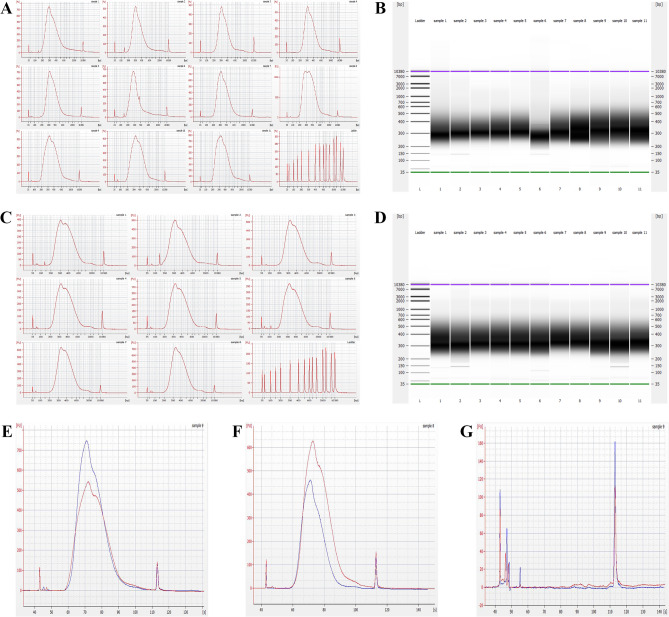
Enzymatic fragmentation final library Quality Control. (**A**) Electropherograms of on-platform (Samples 1–8) and positive control (Samples 9–11) human DNA libraries. The bottom right panel shows a DNA ladder for size comparison. (**B**) Gel image demonstrating fragment size distributions of the human DNA libraries shown in (**A**). (**C**) Electropherograms of on-platform (Samples 1–6) and positive control (Sample 7–8) *E. coli* DNA libraries. The bottom right panel shows a DNA ladder for size comparison. (**D**) Gel image demonstrating fragment size distributions of the *E. coli* DNA libraries shown in (**C**). (**E**) Human DNA positive control (red) and on-platform (blue) library overlay for yield and size distribution comparison. (**F**) *E. coli* DNA positive control (red) and on-platform (blue) library electropherogram overlay for yield and size distribution comparison. (**G**) Manual (red) and on-platform (blue) negative control electropherogram overlay.

Manual positive controls consisting of 50 ng gDNA (*E. coli* and human) were constructed using the enzymatic fragmentation assay intended for WGS. Supplementary Table 3B shows the yield and percent adapter-dimer for each sample and positive control corresponding to the enzymatic fragmentation assay and either human or *E. coli* gDNA input. Electropherograms and gel images further confirm that our positive control libraries pass the criteria in terms of the size distribution (Fig. 8A–D) (average fragment size ~ 350 bp for both human and *E. coli* DNA). Positive control average yield and percent adapter-dimer were 3132.4 $\pm$ 718.14 ng and 0% for the human DNA libraries and 1459.3 $\pm$ 3.54 and 0% for the *E. coli* DNA libraries, respectively (S. Table 3B), consistent with passing criteria. Manual and on-platform negative controls were performed without input DNA to assess reagent and platform contamination. No erroneous peaks were observed; the electropherogram overlays shown in Fig. 8G highlight the presence of a single adapter-dimer peak as expected. Thus, we concluded that the reagents and platform were not contaminated.

**Table 1:** Library preparation efficiency analysis.

Variable Name	Definition	Mechanical fragmentation	Enzymatic fragmentation
$m_{in}$	Input mass (g)	20 ng	50 ng
$\langle l_{bpi} \rangle$	Length of input DNA fragment (bp)	260 bp	50 kbp → average 250 bp fragments
$N_{DNA}$	Number of input DNA molecules	**7.19** $\times$ **10**^**10**^	**1.87** $\times$ **10**^**11**^
$\eta_{lig}$	Ligation efficiency	60%	65%
$N_{AL}$	Number of adapter-ligated DNA molecules	**4.31** $\times$ **10**^**10**^	**1.21** $\times$ **10**^**11**^
$\eta_{wash}$	Total wash step efficiency	73%	77%
$\eta_{PCR}$	PCR efficiency	95%	95%
$N_{PCR}$	Number of adapter-ligated DNA molecules post-PCR	**7.62** $\times$ **10**^**12**^	**2.27** $\times$ **10**^**13**^
$F_{ads}$	Fraction of DNA lost to adsorption	0.05	0.05
$F_{LT}$	Fraction of DNA lost during liquid transfers	0.15	0.15
$N_{out}$	Number of DNA molecules in output (final library)	**4.47 ** $\times$ **10**^**12**^	**1.41 ** $\times$ **10**^**13**^
$m_{out}$	Theoretical total output DNA mass	**809 ng**	**2360 ng**
$l_{A}$	Length of 1 adapter (bp)	70	75
$V_{RsB}$	Final resuspension buffer volume	33 µL	22 µL
$C_{f}$	Theoretical final library concentration	**24.5 ng/µL**	**107 ng/µL**

Significant values are in [bold].

#### Libraries from *Escherichia coli* bacterial DNA

We evaluated the size distribution of libraries generated from our device and compared them to positive controls to gauge the effectiveness of our method for preparing sequencing-ready *E. coli* DNA libraries. Figures 7C and 8C show broad bands in the ~ 200–1000 bp region, corresponding to the size distribution of adapter-bearing, indexed, DNA fragments. The average yield and % adapter-dimer for on-platform *E. coli* libraries generated by the mechanical and enzymatic fragmentation assays were 610.25 $\pm$ 128.93 ng and 0.44 $\pm$ 0.0041%, and 960.97 $\pm$ 176.47 ng and 0.28 $\pm$ 0.0056%, respectively. An additional peak at ~ 150 bp corresponds to adapter dimers that formed during ligation^16^. The lack of erroneous peaks highlights the success of the post-PCR SPRI bead purification step in removing primer dimers. Indexed fragments mainly fell within the desired 200–1000 bp size range, though a small, broad hump in the baseline past 1000 bp for the enzymatic fragmentation assay (Fig. 8C) suggests that some high molecular weight products remained post-fragmentation. However, the presence of this same hump in the positive controls (Fig. 8C, Samples 7–8) signifies the success of our method in replicating results obtained from the gold-standard practice. Conversely, the broad peak past 1000 bp is also present in the mechanical fragmentation assay electropherograms (Fig. 7C), but only for the libraries obtained on-platform. This could be a result of slightly more efficient manual purification steps, including bead-drying and alcohol wash removal, compared to on-platform purification in which the broad hump could be indicative of leftover enzymes or protein. Figure 7F shows an electropherogram overlay of *E. coli* positive control (red) and on-platform (blue) libraries generated by the mechanical fragmentation assay, while Fig. 8F shows an electropherogram overlay of *E. coli* positive control (red) and on-platform (blue) libraries generated by the enzymatic fragmentation assay, for visual comparison.

#### Libraries from human DNA

Human DNA libraries obtained via our automated method were evaluated for their size distribution. Again, the fragment size distribution fell within the desired ~ 200–1000 bp region (Figs. 7A, 8A). Adapter-dimer can be seen at ~ 150 bp, but no significant primer-dimer is present. The average yield and % adapter-dimer for on-platform human libraries generated by the mechanical and enzymatic fragmentation assays were 677.20 $\pm$ 105.62 ng and 0.51 $\pm$ 0.0022%, and 2204.9 $\pm$ 493.78 ng and 0.23 $\pm$ 0.0034%, respectively. Similar to the *E. coli* libraries, the electropherograms of the human libraries produced by the enzymatic fragmentation assay show a trailing edge past 1000 bp, highlighting the presence of high molecular weight DNA fragments and possible gDNA contamination remaining from the fragmentation reaction (Fig. 8A). Slight high molecular weight DNA contamination is expected as we did not perform double-sided size selection for any of the human or *E. coli* libraries produced by the enzymatic fragmentation assay and therefore only removed small fragments including adapter and primer-dimers in both purification steps. Like the *E. coli* libraries produced by the mechanical fragmentation assay, the human libraries also show a small, broad hump past 1000 bp, while the positive controls do not. Therefore, we can say that this broad hump is not dependent on the input sample but perhaps a function of purification step precision and bead binding efficiency. Figure 7E shows an electropherogram overlay of human positive control (red) and on-platform (blue) libraries generated by the mechanical fragmentation assay, while Fig. 8E shows an electropherogram overlay of human positive control (red) and on-platform (blue) libraries generated by the enzymatic fragmentation assay, for visual comparison. It is important to note that all final libraries produced via enzymatic fragmentation (*E. coli* and human) were diluted appropriately to be within the concentration range of the Bioanalyzer dsDNA High Sensitivity Assay (necessary due to 50 ng input and 8 PCR cycles) (S. Table 3B).

#### Library preparation efficiency analysis

We performed a library preparation efficiency analysis to further quantify and compare libraries produced via our automated method to those generated via manual preparation. All library preparation protocols mandate an input mass (in g) of DNA, $m_{in}$, for optimal efficiency and quality of sequence-able fragments. Hence, the number of input DNA fragments, $N_{DNA}$, are represented by

4 $$N_{DNA} = \frac{{m_{in} N_{A} }}{{650\langle l_{bpi} \rangle }},$$

where *N*_*A*_ is the Avogadro’s number, $$\langle l_{bpi} \rangle$$ is the average length of the input DNA, and 650 g/mol is the molar mass of a base pair. Proceeding step-wise throughout the library preparation assay, the efficiency of ligation $$\eta_{lig}$$ must be taken into account before bead purification efficiency to obtain the number of adapter-ligated DNA fragments present post-ligation ($$N_{AL} )$$.

5 $$N_{AL} = \eta_{lig} N_{DNA}$$

The efficiency of the first purification (wash 1) can be attributed to an assembly of bead binding efficiency ($\eta_{bb} )$, bead retention efficiency ($\eta_{br} )$ owing to bead loss throughout the assay, and bead elution efficiency $(\eta_{e} )$: $\eta_{wash} = \eta_{bb} \eta_{e} \eta_{br} .$ However, for the sake of simplicity, we will refer to the overall wash efficiency $\eta_{wash}$.

The number of post-PCR sequence-able DNA fragments $\left( {N_{PCR} } \right)$ depend on the efficiency $(\eta_{PCR} ),$ of $\left( {n = 8} \right)$ PCR cycles and ($\eta_{wash} )$, as described below.

6 $$N_{PCR} = 2^{n} \eta_{wash} \eta_{AL} \eta_{PCR} N_{AL}$$

The post-PCR purification step generates the same efficiency as the first purification step since both are the same protocol with different volumes. Therefore, the number of sequence-able DNA fragments in the final library ($$N_{out} )$$ is estimated via

7 $$N_{out} = N_{PCR} \eta_{wash} \left( {1 - F_{ads} } \right)\left( {1 - F_{LT} } \right),$$

where $F_{ads}$ and $F_{LT}$ represent the fraction of DNA lost to adsorption and during liquid transfers, respectively. The intent of our method is to keep these fractions close to zero. To better quantify and analyze library preparation efficiency, we convert the number of output DNA molecules to sequence-able DNA mass ($m_{out}$) in g, by accounting for the length and subsequent mass change of the DNA fragments post-adapter ligation as

8 $$m_{out} = 650\frac{{N_{out} }}{{N_{A} }}\frac{{\langle l_{bpi} \rangle }}{{{\langle l_{bpi} \rangle + 2\langle l_{A} \rangle}}\langle l_{bpi} \rangle}$$

Here, $$l_{A}$$ is the length of a single adapter in bp. Final library concentration in ng/µL ($$C_{f} )$$ can be determined by dividing the output mass by the volume of the resuspension buffer.

9 $$C_{f} = \frac{{m_{out} }}{{V_{RsB} }}$$

Table 1 lists the calculated and constant values for each of the mentioned variables for both assays. Ligation and PCR efficiency values are founded on previously established data^35–37^. We assume that fragmentation and ERA reaction efficiencies are close to 100%, considering the minimal presence of unfragmented gDNA in final libraries (Fig. 7).

Our calculations demonstrate that the majority of the input DNA is lost during the ligation and purification steps. Adapter-ligation reactions are known to be sensitive and highly reliant on the specific ligase enzyme and critical mixing of the ligase-adapter-DNA reaction^38^. Ligation efficiency can range anywhere from 3 to 100%, with most ligases falling in the 15–40% range^39^. Our automated adaptation of the ligation reaction accounts for its high viscosity and sensitive nature, as discussed above. Slow aspiration speeds allow for sufficient mixing without facilitating adapter-dimer formation, so ligation efficiency is presumed to be comparable on- and off-deck as the reaction conditions are optimized in both cases.

Purification step efficiency is a function of SPRI bead binding adsorption–desorption kinetics, a reaction largely dependent on bead formulation, including surface-coating consistency,texture, and buffer composition. SPRI beads are generally made of polystyrene covered in a layer of magnetite and positively charged carboxyl groups, allowing negatively charged DNA to bind to the beads^18^. If the bead’s surface coating is uneven, DNA will not bind uniformly, and fragments will be lost during the purification steps, decreasing wash step efficiencies. Bead loss (discussed in efficiency analysis in terms of bead retention) via supernatant aspiration is a parameter unique to our automated method and can be regarded as negligible when performing library preparation manually. Though our approach produces high-quality DNA libraries, bead loss is more significant on-platform as a result of limited cannula tip motion in the y-direction and bead adherence to the cartridge tubing. This challenge is addressed via carefully crafted mixing techniques that take advantage of fluid flow and convective mixing in the vertical cannula tips to maximize DNA-bead adsorption and elution, thereby enhancing final library yield.

PCR is well-established in literature as highly efficient. We assumed PCR efficiency to be the same across both assays and positive controls. DNA adsorption to the cartridge tubing is another parameter unique to our automated platform, as it is negligible in the conventional manual approach. Though surface passivation greatly reduces DNA adsorption rates, a small fraction (~ 5%) is lost when liquid is transferred inside the cartridge tubing.

**Figure 9:**
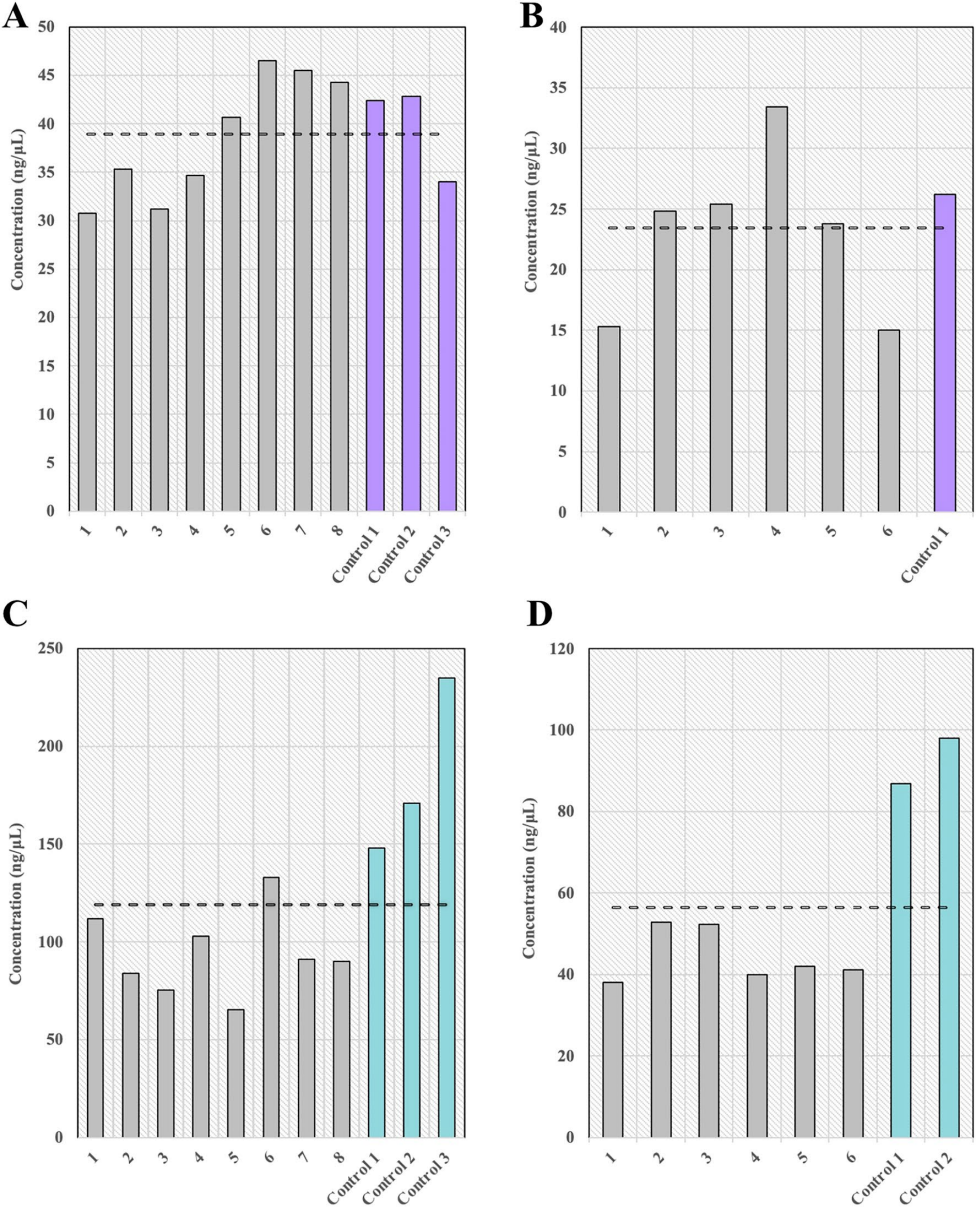
Positive control and on-platform final library concentrations in ng/µL for (**A**) 20 ng input human DNA libraries produced by the mechanical fragmentation assay, (**B**) 20 ng input *E. coli* DNA libraries produced by the mechanical fragmentation assay, (**C**) 50 ng input human DNA libraries produced by the enzymatic fragmentation assay, and (**D**) 50 ng input *E. coli* DNA libraries produced by the enzymatic fragmentation assay. Dashed lines represent the average library concentration across samples and positive controls for each group. Note that all Qubit concentration measurements have a standard error of 12%.

Figure 9 provides a visual summary of the final library concentrations for each of the initial four sample groups. On-platform libraries produced by the mechanical fragmentation assay averaged 38.6 $\pm$ 6.42 ng/µL (CV of 0.166) and 23.0 $\pm$ 6.94 ng/µL (CV of 0.302) for human and *E. coli* DNA, respectively, compared to 39.7 $\pm$ 4.97 ng/µL (human) (CV of 0.125) and 26.2 ng/µL (*E. coli*) positive controls (Fig. 9A,B). The theoretical final library concentration was calculated as ~ 24.5 ng/µL (Table 1). Positive control and on-platform final library concentrations for the human DNA libraries differed by ~ 1 ng/µL, while the *E. coli* libraries differed from positive controls by ~ 3 ng/µL, with slightly higher positive control concentrations in both cases, as expected. The average on-platform *E. coli* final library concentration fell within ~ 1.5 ng/µL of the calculated theoretical value, while the average on-platform human final library concentration was ~ 38% higher than calculated. It is important to note that the Qubit Flex Fluorometer has a known error of $\pm$ 12%^39^, and is a potential causative agent of the difference between measured and calculated concentrations. On-platform libraries produced by the enzymatic fragmentation assay averaged 94.2 $\pm$ 21.5 ng/µL (CV of 0.228) and 44.4 $\pm$ 6.47 ng/µL (CV of 0.146) for human and *E. coli* DNA, respectively, compared to 185 $\pm$ 45.1 ng/µL (human) (CV of 0.244) and 92.4 $\pm$ 7.92 ng/µL (*E. coli*) (CV of 0.086) positive controls (Fig. 9C,D). The theoretical final library concentration was calculated as ~ 107 ng/µL (Table 1). Positive control and on-platform final library concentrations for the human DNA libraries differed by ~ 90 ng/µL, while the *E. coli* libraries differed from positive controls by ~ 48 ng/µL, with more concentrated positive controls in both cases, as expected. The average on-platform *E. coli* final library concentration was ~ 12% lower than calculated, while the average on-platform human final library concentration was ~ 42% higher than calculated.

**Figure 10:**
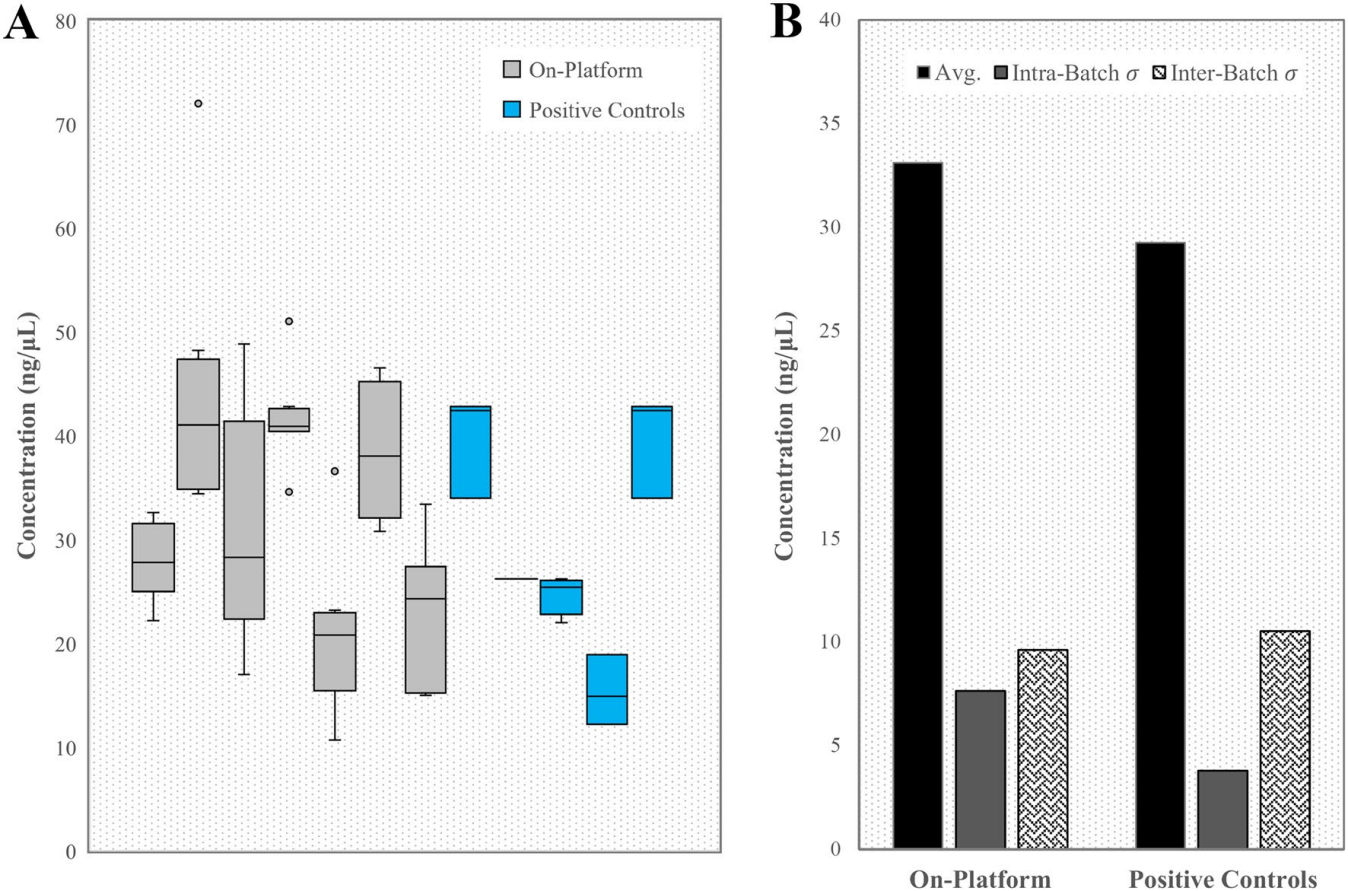
Final library concentrations across multiple sample preparation sessions for mechanical fragmentation assay with assorted DNA input. (**A**) Automated plates compared to several batches of manually prepared positive controls. Each box represents a separate plate (for on-platform samples) or separate batch of manual samples (for positive controls). (**B**) Average library concentration across 54 on-platform samples and 14 manual positive controls. Sample-to-sample yield standard deviation, as well as standard deviation between average batch performances were determined to examine consistency between samples and between platform runs.

To further examine on-platform performance and consistency, we gathered final library concentrations from 7 different on-platform mechanical fragmentation assay runs conducted with mixed human and *E. coli* DNA, as well as 4 different batches of manual positive controls (Fig. 10A). All samples prepared passed sequencing criteria. On average, on-platform samples had a final concentration of 33.1 ng/µL. Average standard deviation across samples from the same plate was 7.64 ng/µL, while standard deviation across average plate concentrations was 9.61 ng/µL (Fig. 10B). Manual positive controls averaged 29.3 ng/µL, with an average intra-batch standard deviation of 3.80 ng/µL and a batch-to-batch standard deviation of 10.5 ng/µL (Fig. 10B).

Final library concentration for the mechanical fragmentation assay is consistently comparable to manually prepared libraries, with similar batch-to-batch variation. Consistency in final library concentration is an indication that PCR amplification occurs reliably on the platform, achieving results comparable to a standard thermal cycler. The variability between concentrations of libraries produced in the same run can be attributed to inconsistent adsorption throughout capillaries in the same cartridge (due to uneven surface morphology contributing to variable adsorption rates and ultimately uneven surface passivation), and variable liquid transfer efficiency between cannula tips (S. Figure 2). Any differences in final concentrations observed between on-platform and positive control libraries is due to the difference in purification step efficiencies on and off-deck (Table 1), as well as DNA adsorption, and liquid transfer efficiency compared to manual pipetting. We further note that, in Fig. 9, the contrast between mechanical fragmentation and enzymatic fragmentation is quite interesting. Reduced yield in the on-platform libraries is primarily observed when enzymatic fragmentation is performed, suggesting that the Frag/AT step of the assay is less efficient on-platform. This can likely be attributed to adsorption of enzyme on the cartridge tubing surface, even after surface passivation. Inconsistency in human vs. *E. coli* DNA final library concentrations produced via the same assay with the same amount of input nucleic acid material is accredited to differences in initial DNA dilutions, including pipetting and quantification discrepancies. Despite the inconsistencies described above, it is critical to realize that both on-platform and positive control libraries pass the criteria for sequencing success in terms of concentration, as the minimum sequence-able concentration is 5 ng/µL^40^. Overall, our library prep efficiency analysis provides insights into the mechanisms of sample loss within NGS library preparation assays compared with automated adaptations and pinpoints areas of future research, improvement, and optimization. Of course, our analysis is simply an estimate useful for contextualizing experimental data, and calculated final library concentrations are expected to differ from experimentally obtained results simply as a consequence of error and approximation propagation.

### Sequencing of human and *E. coli* DNA

#### Mechanical fragmentation

**Figure 11:**
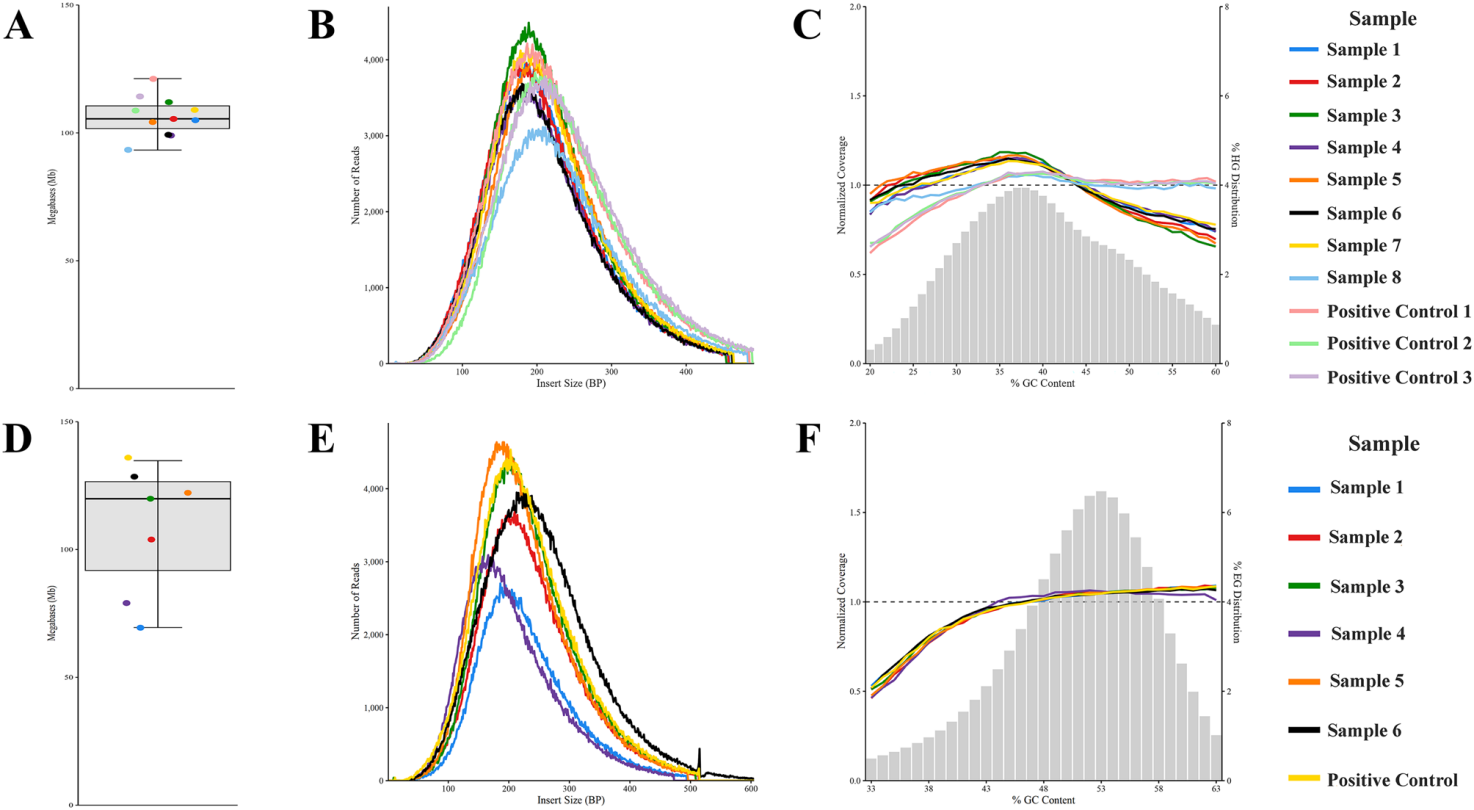
Sequencing data analysis for the mechanical fragmentation assay. Data yield per sample in Mb for (**A**) human DNA input and (**D**) *E. coli* DNA input. Number of reads vs. insert size for (**B**) human DNA input and (**E**) *E. coli* DNA input. Sequencing bias analysis in terms of normalized coverage vs. % GC content for (**C**) human DNA input and (**F**) *E. coli* DNA input.

Libraries produced by the mechanical fragmentation assay were subject to WGS for both human and *E. coli* DNA. As part of our sequencing data analysis, Phred quality scores generated by the Illumina MiniSeq platform were extracted from FASTQ files produced during sequencing. Quality scores are assigned on a logarithmic scale, where $Q = - log_{10} P$ (where P is the probability of a base being incorrectly called)^41,42^. Base calls with Q < 20 (99% base call precision) are considered low quality and a standard indication of poor performance of the sequencing instrument or poor sample quality^43^. Mean Phred (Q) scores were > 36 for all sequencing data generated by the mechanical fragmentation assay, with ~ 99% mean alignment (sequence similarity to a reference genome) for the human DNA reads and ~ 97% mean alignment for the *E. coli* DNA reads (Table 2). Note that Q = 30 corresponds to 99.9% base call precision^44^. No significant difference between on-platform and positive control samples was observed with respect to Phred score or % alignment (Table 2). Data quality was assessed as a measure of data yield and number of reads vs. DNA insert size (Fig. 11A,B, D,E). Total sequencing data yield generated ~ 1,175 $\pm$ 0.25 megabases (Mb) for the human group, averaging ~ 104 $\pm$ 0.25 Mb per sample (Fig. 11A) and ~ 755 $\pm$ 0.25 Mb for the *E. coli* group, averaging ~ 103 $\pm$ 0.25 Mb per sample (Fig. 11D). Positive controls averaged ~ 115 $\pm$ 0.25 Mb for the human group and ~ 135 $\pm$ 0.25 Mb for the *E. coli* group. Figure 11 B,E is a visual representation of the number of sequencing reads vs. DNA insert size in bp for both DNA input groups. Average read length was between 100 and 200 bp for both groups, with the highest number of reads generated from on-platform libraries (~ 11,000 reads for human and ~ 5000 reads for *E. coli*), with human and *E. coli* positive controls falling between 3000–5000 and 3000–4500 reads, respectively (Fig. 11B,E). Sample-to-sample variation in the number of reads vs. insert size is likely due to library pooling and uneven cluster generation due to varying input library concentrations.

**Table 2:** Mean phred (Q) scores and % alignment.

Data group	On-platform samples				Positive controls			
	Average Q Score	Std. Dev	Average % Alignment	Std. Dev	Average Q Score	Std. Dev	Average % Alignment	Std. Dev
Mechanical Fragmentation Human	~ 36.08	$\pm$ 0.47	~ 98.91	$\pm$ 0.06	~ 36.52	$\pm$ 0.31	~ 99.20	$\pm$ 0.00
Mechanical Fragmentation *E. coli*	~ 36.27	$\pm$ 0.41	~ 97.01	$\pm$ 0.06	~ 36.10	$\pm$ 0.72	~ 96.92	$n/a$
Enzymatic Fragmentation Human	~ 36.40	$\pm$ 0.53	~ 98.52	$\pm$ 0.47	~ 36.60	$\pm$ 0.20	~ 99.24	$\pm$ 0.02
Enzymatic Fragmentation *E. coli*	~ 35.71	$\pm$ 0.69	~ 96.57	$\pm$ 0.12	~ 35.92	$\pm$ 0.58	~ 96.58	$\pm$ 0.01

**Figure 12:**
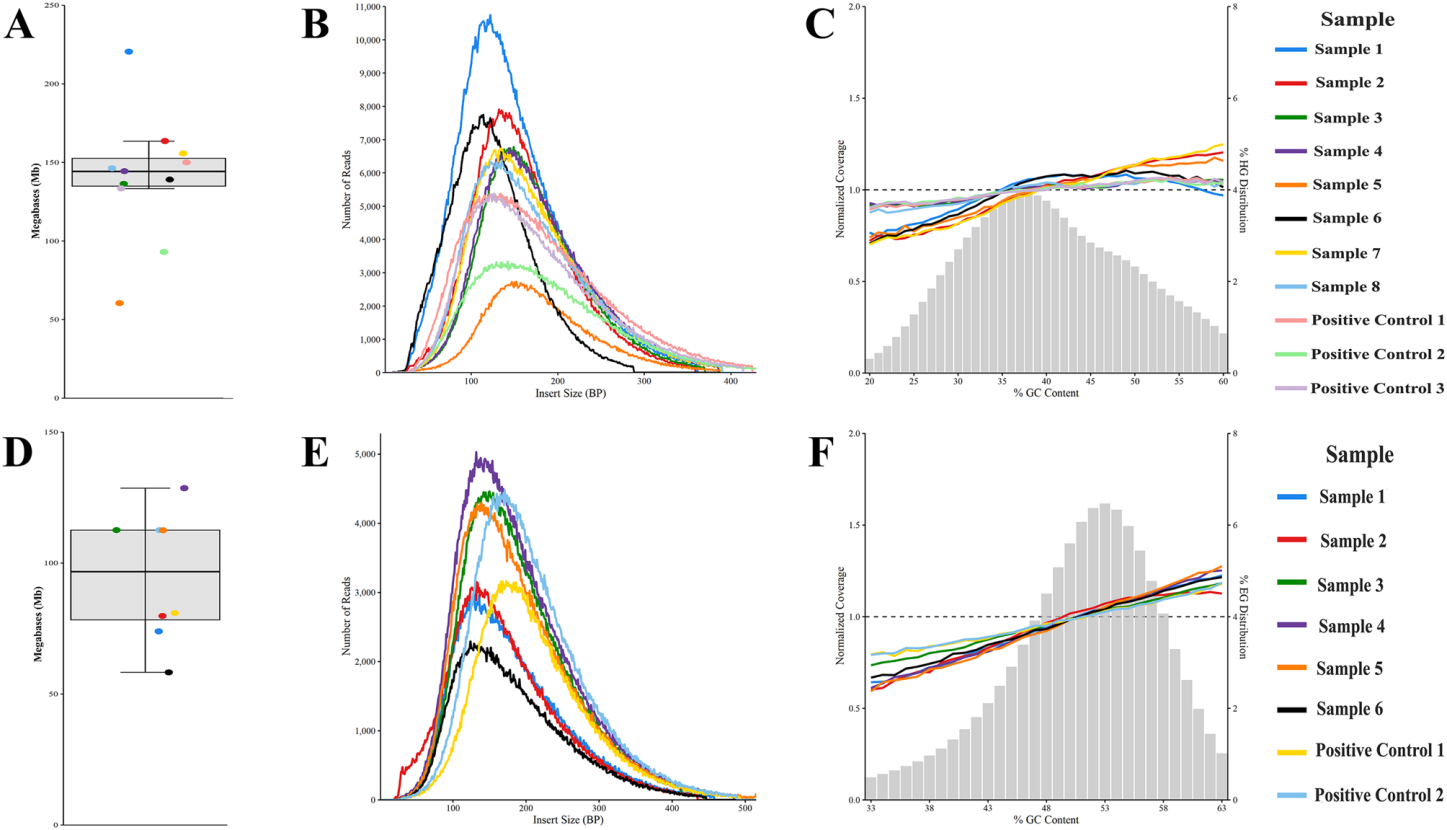
Sequencing data analysis for the enzymatic fragmentation assay. Data yield per sample in Mb for (**A**) human DNA input and (**D**) *E. coli* DNA input. Number of reads vs. insert size for (**B**) human DNA input and (**E**) *E. coli* DNA input. Sequencing bias analysis in terms of normalized coverage vs. % GC content for (**C**) human DNA input and (**F**) *E. coli* DNA input.

Genome coverage analysis was performed to generate normalized coverage vs. % GC content plots. The human and *E. coli* groups show normalized coverage close to 1, with slight underrepresentation of AT-rich regions and overrepresentation of GC-rich regions of the two genomes (Fig. 11C,F). However, both on-platform samples and positive controls have acceptable distributions, eliminating our platform as a perpetuator of sequencing bias. Note that GC bias is, in part, affected by size selection, where slight differences between manual and automated pipetting during bead-based cleanup steps can impact a sample’s bias distribution. We note that on-platform libraries for human DNA in both the mechanical and enzymatic fragmentation assays (Figs. 11C, 12C) exhibit slightly more GC bias than positive controls. This may suggest that the steps common to both workflows, especially the PCR and bead-based cleanup steps, are possible sources of discrepancy between on-platform and manual library construction*.* Still, no observed variation in GC bias between on-platform and manually prepared samples fell outside of passing criteria or became cause for concern.

Mechanical fragmentation (sonication in this case), ERA, and ligation reactions are possible sources of bias present in both on-platform and positive control libraries. The grey bars represent the % GC content distributed across either the human or *E. coli* genome. The bar chart is centered around ~ 38% GC content for human (Fig. 11C) and ~ 53% GC content for *E. coli* (Fig. 11F) DNA, representing the different amounts of GC in each genome (40.9% for human^45^ and 50% for *E. coli*^46^). Plots were generated using Picard, and normalized coverage was calculated by way of the GC bin method in which the reference genome is categorized into bins corresponding to GC content between 20 and 60%, then the number of reads in each GC bin is divided by the average number of reads for all GC bins. Percent GC is calculated based on a set read length of 100 bp known as ‘window-size’.

#### Enzymatic fragmentation

Libraries produced by the enzymatic fragmentation assay underwent WGS for both human and *E. coli* DNA. Mean Q scores were > 35 for all sequencing data produced by the enzymatic fragmentation assay, with ~ 98.7% mean alignment (sequence similarity to a reference genome) for the human DNA reads and ~ 96.5% mean alignment for the *E. coli* DNA reads (Table 2). No significant difference between on-platform and positive control samples was observed with respect to Phred score or % alignment (Table 2). Sequencing generated ~ 1,449 $\pm$ 0.25 Mb of raw data for the human group, averaging ~ 147 $\pm$ 0.25 Mb per sample (Fig. 12A), and ~ 761 $\pm$ 0.25 Mb for the *E. coli* group, averaging ~ 95 $\pm$ 0.25 Mb per sample (Fig. 12D). Positive controls averaged ~ 92 $\pm$ 0.25 Mb per sample for the human group and ~ 97 $\pm$ 0.25 Mb per sample for the *E. coli* group. Average read length was between 100 and 200 bp for both groups (Fig. 12B,E), with on-platform libraries at ~ 11,000 reads for human (Fig. 12B) and ~ 5000 reads for *E. coli* (Fig. 12E), and positive controls falling between 3000–5000 and 3000–4500 reads, respectively. Again, sample to sample variation is due to varying input library concentrations.

Both the human and *E. coli* groups demonstrate normalized coverage close to 1, with an underrepresentation of AT-rich regions and an overrepresentation of GC-rich regions, as expected (Fig. 12C,F). Positive controls have comparable coverage distributions, indicating negligible sources of bias generated by our instrument. However, enzymatic fragmentation is an added overarching source of potential bias in this case, as fragmentation enzymes are notorious for inconsistent fragmentation among different % GC regions of the genome^47^. The grey bar chart is centered around ~ 38% GC content for human (Fig. 12C) and ~ 53% GC content for *E. coli* (Fig. 12F) DNA, representing the different amounts of GC in each genome, and aligns with known %GC content for the human and *E. coli* genomes.

By simplifying various steps in the library preparation and using reasonably low quantities of input DNA, our system could be employed for surveillance and identification of potential disease outbreaks at early stages, isolating sources of a pathogen by molecular genotyping of bacterial isolates^48,49^.Our platform has the potential for use across many small laboratories, enabling rapid sequencing-based discovery of outbreaks.

## Conclusions

As NGS library preparation remains a significant bottleneck to the entire genomic research and clinical community, it is essential that innovative engineering technologies automate the process and meet standards for accurate sequencing and diagnostic screening. Our design innovation presents a platform that combines all elements of DNA NGS library preparation by employing simplified micro and macroscale geometries to efficiently perform purification, reaction heating and mixing, and fluidic transport. DNA libraries prepared using our platform met the same standard as libraries prepared manually in terms of coverage bias, yield, and fragment size. Our scientific approach uniquely integrates automation into applications in areas such as precision medicine, genomic surveillance, and antibiotic resistance tracking. Our method is ideal for smaller laboratories where high-throughput robotic liquid handlers are not a feasible option for automating library preparation. By adopting our method, the research community is expected to save time and resources as well as more effectively process clinical samples. Select work in the field, such as performed by Shi, C. et al., has shown promising results for clinical applications of low-throughput NGS library preparation automation solutions^50^. Their cassette-dependent system, though different in design from our unified cartridge-plate system, was used to detect BRCA1 and BRCA2 mutations with high accuracy, high precision, and minimal cross-contamination, demonstrating the utility of platforms of this format in a clinical research setting. As part of future studies, we look forward to testing our device with clinical samples and further examining its ability to prevent cross-contamination.

## Experimental procedures and materials

### Library preparation methods

We applied our method to two library preparation kits, one utilizing mechanical DNA fragmentation (sonication) and the other utilizing enzymatic fragmentation.

#### Mechanical fragmentation

We constructed positive controls and on-platform libraries using the NEXTFLEX Rapid DNA-Seq Kit 2.0 for Illumina Platforms (Perkin Elmer Inc., Waltham, MA) to assess mechanical fragmentation library preparation. The manufacturer-issued protocol was used to construct all mechanically fragmented positive controls. Fragmented DNA, water, ERA buffer, and ERA enzyme were combined and incubated for 30 min at 20 °C and 30 min at 65 °C using the MiniAmp™ Thermal Cycler (ThermoFisher Scientific, Waltham, MA). Adapters were diluted with a 1:40 dilution and then homogenized with the ERA mixture, ligase enzyme, and ligase buffer. The mixture was incubated for 15 min at 20 °C, then purified with cleanup beads and 70% IPA (isopropyl alcohol) per the kit instructions. DNA was eluted in resuspension buffer, and the supernatant was combined with primer and PCR master mix. 8 cycles of PCR were performed, with an initial denaturation of 30 s at 98 °C, denaturation for 15 s at 98 °C, annealing for 30 s at 65 °C, and extension for 30 s at 72 °C, with a final extension for 2 min at 72 °C. A second wash was performed with cleanup beads and 70% IPA per kit instructions to yield the final library of adapter-ligated DNA.

#### Automated adaptation

For the on-platform adaptation of the protocol, a pretreatment solution was added for surface passivation of the cartridge tubing, comprised of ERA buffer from the library preparation kit, 20 mg/mL BSA (New England Biolabs, Ipswich, MA), 10% Tween-20 (Bio-Rad Laboratories, Hercules, CA), 30 ng/μL oligo (Integrated DNA Technologies, Coralville, OH), and nuclease-free water (Integrated DNA Technologies, Coralville, OH). Adapters and primers (2 μL each) were combined with 8 μL of water when placed in their respective wells to prevent evaporative loss and liquid handling errors. We consider a total volume of 10 μL in wells of the plate to be a large enough volume for consistent plate preparation, while not exceeding the volume allowed at the heating element for PCR. Positive controls were performed with added water to ensure there were no negative results from ligation and PCR reaction dilution. 28 μL of resuspension buffer was used for elution at the end of the post-ligation cleanup and 35 μL at the end of the post-PCR cleanup. All other volumes remained the same in the on-platform adaptation. All temperatures and incubation times were consistent with the manual protocol (S. Table 1). Note that room temperature and humidity are slightly variable from run-to-run of the platform, however we have not observed any negative effects from these variations.

#### Enzymatic fragmentation

Additional positive controls and on-platform libraries were constructed with the Library Preparation Enzymatic Fragmentation Kit 2.0 (Twist Bioscience, South San Francisco, CA) to assess enzymatic fragmentation library preparation. The manufacturer-issued protocol was used to construct all enzymatically fragmented positive controls. Genomic DNA, water, frag/AT buffer, and frag/AT enzymes were combined and incubated for 20 min at 37 °C, then 30 min at 65 °C in a thermal cycler. Adapters and ligation master mix were added to the fragmentation mixture. The mixture was incubated for 15 min at 20 °C, then purified with purification beads and 70% IPA per the kit instructions. DNA was eluted in nuclease-free water, and the supernatant was combined with primer and PCR master mix. 8 cycles of PCR were performed, with an initial denaturation of 45 s at 98 °C, denaturation for 15 s at 98 °C, annealing for 30 s at 60 °C, and extension for 30 s at 72 °C, with a final extension for 1 min at 72 °C. A second wash was performed with purification beads and 70% IPA per kit instructions to yield the final library of adapter-ligated DNA.

#### Automated adaptation

For the on-platform adaptation of the protocol, we used the same blocking pretreatment as the mechanical fragmentation assay. Adapter was combined with 5 μL water to bring total well volume to 10 μL when placed in the reagent plate. No water was added to primer wells, as that volume was already 10 μL. 23 μL of resuspension buffer was used for elution at the end of post-ligation cleanup and 27 μL at the end of post-PCR cleanup. All other volumes remained the same in the in-platform adaptation. All temperatures and incubation times were consistent with the manual protocol (S. Table 1).

### Nucleic acid starting material

20 ng total (concentration 2 ng/μL) of fragmented *Escherichia coli* (*E. coli*) DNA (average 280 bp) and fragmented human DNA (Female, average 260 bp, Promega, Madison, WI) obtained from Bioo Scientific (Austin, TX) were input into the mechanical fragmentation assay using the NEXTFLEX Rapid DNA-Seq Kit 2.0 for Illumina Platforms. Human and *E. coli* genomic DNA was fragmented using the Covaris E210 Focused-ultrasonicator (Covaris, Woburn, MA).

*E. coli* gDNA (Thermo Fisher Scientific, Waltham, MA) and Human gDNA (Mixed, 50 kbp, Promega, Madison, WI) were input into the enzymatic fragmentation assay using the Library Preparation Enzymatic Fragmentation Kit 2.0. The Twist Library Preparation Enzymatic Fragmentation Kit 2.0 assay required 50 ng total of starting nucleic acid material per the recommendations in the protocol.

### Library quantification and quality control

All starting material and final libraries were quantified using an 8-channel Qubit Flex Fluorometer and the dsDNA High Sensitivity (HS) assay (Invitrogen, Waltham, MA). Input DNA and final libraries were assessed for quality control using the High Sensitivity DNA assay for dsDNA on an Agilent 2100 Bioanalyzer instrument (Agilent Technologies, Santa Clara, CA). Bioanalyzer and Qubit input DNA library concentrations ranged from 5 to 50 ng/µL. Libraries were diluted appropriately to concentrations within the range of the Bioanalyzer High Sensitivity DNA assay prior to chip loading and the HS Qubit assay prior to quantification.

### Mechanical device assembly

Our device utilizes air displacement to achieve accurate reagent aspiration and dispensation. Eight parallel lanes displace liquid via a unified motion of eight syringes (Model 81,320, Hamilton Company, Reno, NV) connected to a horizontal linear rail (SLW-BB-1040-06-100, igus Inc., Cologne, Germany). Airlines connect to a single-use custom cartridge (3D printed) comprised of flexible polymeric tubing (Flexelene brand, Eldon James, CO, USA) and joined to 16 cannula pipette tips (Revvity, Waltham, MA) that insert into the wells of a 384-well reagent plate in two parallel columns of 8. Cannula tips are secured to a vertical linear rail (igus Inc., Cologne, Germany) for movement along the z-axis. The reagent plate is inserted onto a horizontal aluminum frame (machined at Brown University lab), and attached to a linear rail (igus Inc., Cologne, Germany) for movement along the x-axis. A bar magnet (BZ084, K&J Magnetics, Pipersville, PA) moves along the z-axis to press against the reagent plate (269,390, ThermoFisher Scientific, Waltham, MA) during bead pelleting steps. All components move along their respective linear rails via step motor motion control.

The polymeric tubing of the cartridge inserts directly into a grooved Peltier heating element (custom design, machined at Brown machine shop), held in place with a detachable insulated door (custom design, machined at Brown machine shop. A thermocouple (Digi-Key Electronics, Thief River Falls, MN) epoxied within a segment of cartridge tubing and fixed to the heating element allows for real-time logging of thermal data during thermal cycling. Valves are present on both sides of the heating element with the freedom to move along the y-axis (custom design, machined at Brown machine shop). The valves press firmly against the metal PCR door to seal samples inside the polymeric tubing and prevent vapor escape during heating processes. Proper maintenance of pressure, as demonstrated by low leak rates found in Supplementary Material (S. Figure 3).

### Device consumables

The disposable cartridge is single-use and therefore replaced after each preparation of 8 libraries to avoid contamination and ensure accurate liquid handling. The cartridge consists of 16 10 µL pipette tips press-fit without dead volume into a custom cannula boat that fits firmly into the spring-loaded z-axis clamp. Eight separate lanes of flexible polymeric tubing interface with the pipette tips to directly connect them to the device airlines and, subsequently, the syringe pumps via tightly sealed O-rings. Each lane contains a Y-junction to allow for fluid displacement in two separate segments of tubing and subsequent cannula tips situated in parallel columns of eight. Fluid volumes move through the cannula and tubing via Hagen-Poiseuille flow. Consistent cartridge manufacturing is key to the functional operation of the platform. Consequently, each cartridge is subjected to a strict quality control test, where pipette tip alignment, vertical positioning, and fluid aspiration and dispensation are evaluated in a sterile environment.

Every new library preparation (use of the device) requires a new, sterile Thermo Scientific™ Nunc™ DeepWell™ 384-Well Plate (Thermo Fisher Scientific, Waltham, MA). Reagents are loaded into specific columns of the plate by the user, so the platform can transfer them appropriately throughout each assay. Every other row of the plate corresponds to the preparation of a separate indexed library, which allows for successful multiplexing. Plates are centrifuged briefly prior to loading of 70% isopropyl alcohol (IPA) used for the wash steps, then sealed with a 384-well pierceable plate seal (Azenta Life Sciences, Wotton, England).

### Scripting resources

Python was chosen as the main scripting language for assay development, software, and firmware on the platform. The majority of scripting functions are defined in a single master script that sets device landmarks in terms of pump rotations and establishes a framework for mechanical feedback on-platform. The master script translates from pump rotations to linear movement along each axis, allowing for a more user-friendly assay development process. The script contains arrays of column positions for each of the two cannulae, as well as classes for speeds and various consumable reagent plates. Functions exist for motor homing, logging of procedure execution and regulation, setting the temperature of the heating element, generating thermal data for post-run analysis, and refined, customizable X-plate, z-axis, and pump movement.

Assay development occurs in supplementary scripts that import functions from the master script. Each liquid displacement function contains parameters for cannula being used, column number on the plate, speed of pump movement, and, in some cases, a quantity of liquid to be aspirated or dispensed. There are methods defined for mixing at various intensities, with a number of loop repetitions delineated locally in the supplementary scripts. For instance, to resuspend a bead pellet during a library wash step, the assay developer might first use the “move_all” function to transfer resuspension buffer to the column with the bead pellet, then use a de-peletting mixing technique with 40 repetitions to homogenize the SPRI beads and resuspension buffer. Each step of an assay protocol is achieved through these fundamental building blocks; subsystems are called on sequentially to manipulate reagents and heat boluses to desired temperatures. All supplementary scripts are stored in an assay development repository, hosted and managed via Bitbucket (Atlassian, Sydney, Australia).

### Sequencing and data analysis

All samples were sequenced using the MiSeq System (Illumina, San Diego, CA). Data analysis was performed using Trimmomatic^51^, Bowtie2^52^, Samtools^53^, Picard^54^, and custom bash and R scripts. R functions used to generate figures were ggplot2, reshape2, tidyverse, scales, pals, RColorBrewer, and gridExtra.

## Supplementary Information

Supplementary Information.

## Data Availability

The datasets generated and analysed during the current study are available in the NCBI Sequence Read Archive (SRA) repository, https://www.ncbi.nlm.nih.gov/bioproject/PRJNA935804.
