# Supplementary material for: Leveraging the fundamentals of heat transfer and fluid mechanics in microscale geometries for automated next-generation sequencing library preparation
Source: Sci Rep. 2024 May 31;14:12564. doi: 10.1038/s41598-024-63014-x (PMC11637099; doi:10.1038/s41598-024-63014-x)

Leveraging the fundamentals of heat transfer and fluid mechanics in microscale geometries for automated Next-Generation Sequencing library preparation

Olivia Ott*, Sabrina Tolppi*, Jennifer Figueroa Cruz, Khaliun Myagmar, Khulan Unurbuyan, Anubhav Tripathi**

Center for Biomedical Engineering, School of Engineering, Brown University

[*These authors contributed equally.]
[**To whom correspondence may be addressed. E-mail: anubhav_tripathi@brown.edu]

SUPPLEMENTARY MATERIAL

Leveraging the fundamentals of heat transfer and fluid mechanics in microscale geometries for automated Next-Generation Sequencing library preparation

Supplementary Table 1: Variables and values corresponding to Fig. 4. Mixing and aspiration velocities for each step can also be found in S. Table 1 as additional information for Fig. 4.

S. Table 1A

T_{pt}	75°C
t_{pt}	10 min
P_1	1.90 atm
V_1	50 µL
C_{pt}	[BSA] = 0.4 µg/µL; [Oligo] = 0.6 ng/µL; [Tween-20] = 1.1 ng/µL; [ERA Buffer]
C_w	[BSA] = 0.105 µg/µL; [Oligo] = 0.158 ng/µL; [Tween-20] = 4.316 ng/µL; [ERA Buffer] in waste well
v_{mix}	16.7 µL/s
v_{asp}	4.32 µL/s

S. Table 1B

	**Mechanical Fragmentation**	**Enzymatic Fragmentation**
C_i	[sheared DNA] = 0.4 ng/µL<br>[ERA buffer]<br>[ERA enzyme]	[gDNA] = 1 ng/µL<br>[Frag/AT buffer]<br>[Frag/AT enzymes]
T_f	N/A	37°C

t_2	N/A	20 min
T_{era}	65°C	65°C
t_3	35 min	30 min
P_1	1.90 atm	1.90 atm
V_2	50 µL	50 µL
C_a	[sheared DNA] = 0.4 ng/µL<br>[adapter] = 0.012 µM<br>[ligase enzyme]<br>[ligase buffer]<br>[ERA buffer]<br>[ERA enzyme]	[fragmented DNA] = 1 ng/µL<br>[adapter]<br>[ligation master mix]<br>[Frag/AT buffer]<br>[Frag/AT enzymes]
V_3	107.5 µL	80 µL
v_{mix}	16.7 µL/s	16.7 µL/s
v_{asp}	1.67 µL/s	1.67 µL/s

S. Table 1C

	Mechanical Fragmentation	Enzymatic Fragmentation
C_a	[sheared DNA] = 0.4 ng/µL<br>[adapter] = 0.012 µM<br>[ligase enzyme]<br>[ligase buffer]<br>[ERA buffer]<br>[ERA enzyme]	[fragmented DNA] = 1 ng/µL<br>[adapter]<br>[ligation master mix]<br>[Frag/AT buffer]<br>[Frag/AT enzymes]
T_L	Room Temperature	Room Temperature
t_4	20 min	20 min
V_3	107.5 µL	80 µL
C_b	[adapter-ligated DNA]<br>[non-ligated adapter]<br>[ligase enzyme]<br>[ligase buffer]<br>[ERA buffer]<br>[ERA enzyme]	[adapter-ligated DNA]<br>[non-ligated adapter]<br>[ligation master mix]<br>[Frag/AT buffer]<br>[Frag/AT enzymes]
v_{mix}	16.7 µL/s	16.7 µL/s
v_{asp}	1.67 µL/s	1.67 µL/s

S. Table 1D

	Mechanical Fragmentation	Enzymatic Fragmentation

V_3	107.5 µL	80 µL
C_b	[adapter-ligated DNA]<br>[non-ligated adapter]<br>[ligase enzyme]<br>[ligase buffer]<br>[ERA buffer]<br>[ERA enzyme]	[adapter-ligated DNA]<br>[non-ligated adapter]<br>[ligation master mix]<br>[Frag/AT buffer]<br>[Frag/AT enzymes]
V_4	100 µL	60 µL
C_c	[Cleanup beads]<br>[adapter-ligated DNA]<br>[non-ligated adapter]<br>[ligase enzyme]<br>[ligase buffer]<br>[ERA buffer]<br>[ERA enzyme]	[Purification beads]<br>[adapter-ligated DNA]<br>[non-ligated adapter]<br>[ligation master mix]<br>[Frag/AT buffer]<br>[Frag/AT enzymes]
V_5	~205 µL	~138 µL
V_6	198 µL	200 µL
V_7	~196 µL	~198 µL
t_5	~3 min	~3 min
V_8	28 µL	23 µL
C_d	[Cleanup beads]<br>[adapter-ligated DNA]	[Purification beads]<br>[adapter-ligated DNA]
C_e	[adapter-ligated DNA]	[adapter-ligated DNA]
V_9	27 µL	22 µL
v_{mix}	Alternating between 16.7 µL/s and 76.8 µL/s	Alternating between 16.7 µL/s and 76.8 µL/s
$v_{asp, supernatant}$	3.34 µL/s	3.34 µL/s
$v_{disp, IPA}$	20.0 µL/s	20.0 µL/s

S. Table 1E

	**Mechanical Fragmentation**	**Enzymatic Fragmentation**
C_f	[adapter-ligated DNA]	[adapter-ligated DNA]
V_9	27 µL	22 µL
V_{10}	62 µL	57 µL
C_g	[adapter-ligated DNA]<br>[PCR primers]	[adapter-ligated DNA]<br>[PCR primers]

	[PCR master mix]	[PCR master mix]
T_D	98°C	98°C
T_A	65°C	60°C
T_E	72°C	72°C
t_{cycle}	Initial Denaturation: 30 sec<br>Denaturation: 15 sec<br>Annealing: 30 sec<br>Extension: 30 sec<br>Final Extension: 2 min	Initial Denaturation: 45 sec<br>Denaturation: 15 sec<br>Annealing: 30 sec<br>Extension: 30 sec<br>Final Extension: 1 min
P_1	1.90 atm	1.90 atm
C_h	[amplified DNA]<br>[PCR primers]<br>[PCR master mix]	[amplified DNA]<br>[PCR primers]<br>[PCR master mix]
v_{mix}	16.7 µL/s	16.7 µL/s
v_{asp}	1.67 µL/s	1.67 µL/s

S. Table 1F

	**Mechanical Fragmentation**	**Enzymatic Fragmentation**
V_{10}	62 µL	57 µL
C_h	[amplified DNA]<br>[PCR primers]<br>[PCR master mix]	[amplified DNA]<br>[PCR primers]<br>[PCR master mix]
V_{11}	45 µL	50 µL
C_j	[Cleanup beads]<br>[amplified DNA]<br>[PCR primers]<br>[PCR master mix]	[Purification beads]<br>[amplified DNA]<br>[PCR primers]<br>[PCR master mix]
V_{12}	~105 µL	~105 µL
V_{13}	198 µL	200 µL
V_{14}	~196 µL	~198 µL
t_5	~3 min	~3 min
V_{15}	35 µL	27 µL
C_k	[Cleanup beads]<br>[amplified DNA library]	[Purification beads]<br>[amplified DNA library]
C_l	[amplified DNA library]	[amplified DNA library]

V_{16}	33 µL	25 µL
C_e	[adapter-ligated DNA]	[adapter-ligated DNA]
V_9	27 µL	22 µL
V_{mix}	Alternating between 16.7 µL/s and 76.8 µL/s	Alternating between 16.7 µL/s and 76.8 µL/s
$V_{asp, supernatant}$	3.34 µL/s	3.34 µL/s
$V_{disp, IPA}$	20.0 µL/s	20.0 µL/s

Supplementary Figure 1: Example 384-well plate layout for library preparation, including all pre-loaded reagents.

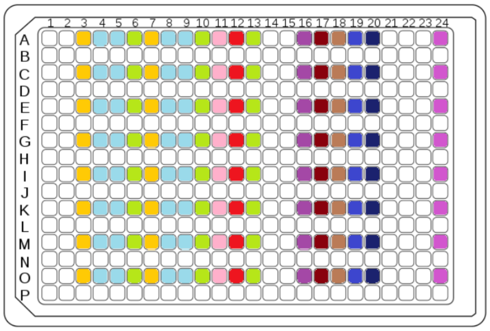

Column 3 - Resuspension buffer
Column 4 - 70% IPA
Column 5 - 70% IPA
Column 6 - 0.5% Tween-20
Column 7 - Resuspension buffer
Column 8 - 70% IPA
Column 9 - 70% IPA
Column 10 - 0.5% Tween-20
Column 11 - Adapter mix
Column 12 - Ligation master mix
Column 13 - 0.5% Tween-20
Column 16 - Pretreatment solution
Column 17 - Cleanup beads
Column 18 - Cleanup beads
Column 19 - Twist UDI primer
Column 20 - Equinox Library Amp Mix (2X)
Column 24 - Fragmentation mix

Liquid handling accuracy: The platform was evaluated for volumetric accuracy during liquid handling. Liquid transfer tests were conducted, with three trials performed for transfers of 3 µL, 5 µL, 10 µL, 20 µL, 50 µL, and 100 µL of water. Final dispensed volume was determined using the density of water and an AL104 Analytical Balance (Mettler Toledo, Columbus, OH).

Volume accuracy of liquid transfers improves with increasing volume, while % CV decreases (Fig 5). The platform has an approximate working range of 10 to 200 µL, which allows it to complete liquid transfers and perform proper mixing of plated reagents. For this volumetric range, % CV remains low, with high accuracy.

Supplementary Figure 2: Volumetric accuracy of the pump assembly. (A) Actual volume dispensed vs. scripted setpoint volumes, and (B) Percent CV vs. scripted setpoint volumes.

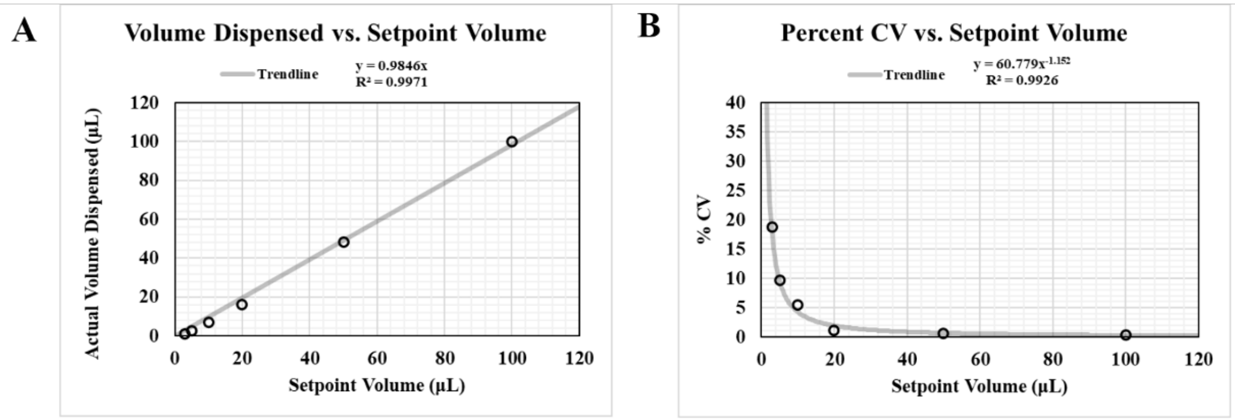

Evaluation of Pump Pressure: The pump's ability to hold pressure is evaluated using a rig with embedded pressure sensors. The rig is placed on the platform with the syringe plungers at position 0, and the second cannula selected by closing the two valves on the other cannula. The syringe assembly moves to position 30 (maximum extension of the plungers) to apply a vacuum for a set period of time. The rig is removed to relieve the vacuum, then replaced. The syringe plungers move to position 0 to apply pressure and are held for a set period of time to monitor leak rates. For a fully functioning platform, leak rates are expected to be insignificant, often no more than 1 psi (0.07 atm) of pressure lost per hour. This process is repeated for the first cannula. The first cannula pressures are negative compared to actual pressures due to the directionality of pressure connections within the set of sensors. The last portion of the test assesses leak rates of individual valves, where the two spikes represent a rapid release of pressure between valve tests (S. Fig. 2).

Supplementary Figure 3: Plot of pressure readings over time during pump pressure evaluation.

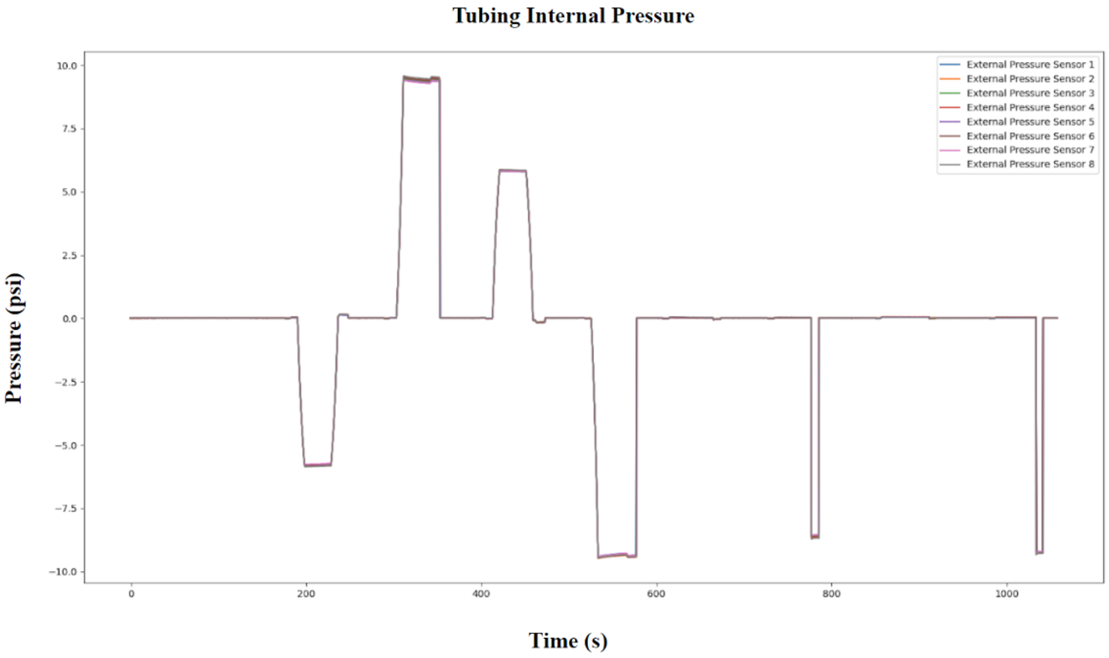

Calculation of Pressure in Fluid Slug During Heating: To quantify the pressure a fluid slug is placed under during heating, the initial and final volume of air between the fluid slug and the syringe plunger were calculated. Boyle's Law states that pressure and volume are inversely proportional, thus $P_1V_1 = P_2V_2$. With an initial volume of 1614.3626 mm^3, a post-pressurization volume of 850.7514 mm^3 (S. Table 2), and an initial pressure of 1 atm, the fluid slug experiences a pressure of approximately 1.90 atm.

Supplementary Table 2: Dead volumes of pump-airline assembly components.

**Component**	**Volume (mm^3)**
Tubing from fluid slug to Y-junction	132.61
Tubing from -junction to cartridge frame	138.55
Interfaces between cartridge and airline	40.43
Airline	296.90
Interfaces between airline and syringes	159.26
Syringe barrel (post-pressurization in parentheses)	846.61 (83.00)
Total (post-pressurization in parentheses)	1614.36 (850.36)

Determination of Library Yield and Percent Adapter-Dimer: Raw data obtained from the Agilent 2100 Bioanalyzer instrument was exported as a .csv file and imported into Microsoft Office Excel. Mass libraries (y in ng) were determined by taking the sum of all concentrations corresponding to a fragment size range from 200 to 1000 bp, multiplying by the final resuspension buffer volume (V_{RsB}), and dividing by the upper limit fragment size ($l_{max} = 1000$ bp). For enzymatic fragmentation the V_{RsB} value is 27 µl for on-deck and 22 µl for the positive manual control process. On the other hand, for mechanical fragmentation, the V_{RsB} value is 35 µl for on-deck and 33µl for the positive manual control process.

$$y = \frac{V_{RsB} \sum_{i=1}^{n} C}{l_{max}}$$

Mass of the adapter-dimer (d) was calculated using the same equation but for a range between 120 and 150 bp for enzymatic fragmentation and 120-144 bp for mechanical fragmentation (owing to different adapter lengths for each assay). Percent dimer was determined by simply dividing the mass dimer by the sum of mass library and mass dimer.

$$\%\, d = \frac{d}{y + d}$$

Mass library, mass dimer, and % dimer were calculated for all libraries, on-deck and positive controls across both assays for Human and E.coli DNA. Samples were considered "passing" (i.e. sequence-able) if they exhibited a mass library ≥ 250 ng and % dimer < 5%. Our method produced libraries with comparable yield and % dimer to positive controls across the board (S. Table 3A, B).

Supplementary Table 3: Bioanalyzer Data and Calculations.
A: Mechanical Fragmentation

Mechanical Fragmentation			
Human sheared DNA	Mass Library/Yield (ng)	Mass Dimer (ng)	% Dimer
1	646.79	3.66	0.56%
2	625.12	3.54	0.56%
3	587.02	1.75	0.30%
4	890.93	7.01	0.78%
5	718.22	2.26	0.31%
6	755.29	6.68	0.88%
7	600.78	1.90	0.32%
8	593.43	2.25	0.38%
Positive Control 1	1047.45	1.82	0.17%
Positive Control 2	965.25	0.92	0.10%
Positive Control 3	723.97	1.84	0.17%
Average	741.30	3.06	0.41%
Std.	159.73	2.03	0.25%
E.coli sheared DNA	Mass Library/Yield (ng)	Mass Dimer (ng)	% Dimer
1	559.26	7.14	1.26%
2	480.08	1.40	0.29%
3	500.04	1.57	0.31%
4	624.23	1.36	0.22%
5	828.81	3.51	0.42%
6	669.05	0.89	0.13%
Positive Control	445.69	0.43	0.10%
Average	586.74	2.33	0.39%
Std.	133.12	2.33	0.40%

B: Enzymatic Fragmentation

Enzymatic Fragmentation				
Human gDNA	Mass Library/Yield (ng)	Mass Dimer (ng)	% Dimer	Dilution
1	296.15	0.88	0.30%	1:10
2	228.43	1.89	0.82%	1:10
3	188.23	0.00	0.00%	1:10
4	245.92	0.00	0.00%	1:10
5	326.59	0.00	0.00%	1:4
6	250.85	1.75	0.69%	1:10
7	492.24	0.00	0.00%	1:4
8	567.04	0.00	0.00%	1:4
Positive Control 1	268.99	0.00	0.00%	1:10
Positive Control 2	274.63	0.00	0.00%	1:10
Positive Control 3	396.10	0.00	0.00%	1:10
Undiluted Average	2457.9	4.11	0.16%	-
Undiluted Std.	679.34	7.45	0.0031%	-
E.coli gDNA	Mass Library/Yield (ng)	Mass Dimer (ng)	% Dimer	Dilution
1	260.33	0.72	0.27%	1:4
2	304.57	4.32	1.40%	1:4
3	263.05	0.00	0.00%	1:4
4	188.27	0.00	0.00%	1:4
5	227.50	0.00	0.00%	1:4
6	197.74	0.00	0.00%	1:4
Positive Control 1	364.20	0.00	0.00%	1:4
Positive Control 2	365.45	0.00	0.00%	1:4

Undiluted Average	1085.6	2.52	0.21%	-
Undiluted Std.	274.70	6.05	0.0049%	-

Final Library Quantification and Percent Error: To assess experimental concentration in terms of theoretical concentration, calculate percent error, and quantify libraries for sequencing, concentration values were obtained for each library under the four conditions (Human vs. E. coli DNA, mechanical fragmentation vs. enzymatic fragmentation) using a Qubit Flex Fluorometer. There is some variability between the concentrations of libraries produced in the same run, which can be partially attributed to Qubit's 12% error rate, as well as inconsistent adsorption throughout tubes in the same cartridge (due to uneven surface passivation), and variable liquid transfer efficiency between cannula tips. Calculated standard deviations quantify this variance between (S. Table 4).

Supplementary Table 4: Final library concentrations.

A: Mechanical Fragmentation

Mechanical Fragmentation			
Human sheared DNA		E.coli sheared DNA	
Sample	Concentration (ng/µL)	Sample	Concentration (ng/µL)
1	30.7	1	15.3
2	35.3	2	24.8
3	31.2	3	25.4
4	34.6	4	33.4
5	40.7	5	23.8
6	46.5	6	15.0
7	45.5	Positive Control	26.2
8	44.3	Average	23.4
Positive Control 1	42.4	Std.	6.46
Positive Control 2	42.8		
Positive Control 3	34		

Average	38.9
Std.	5.84

B: Enzymatic Fragmentation

Enzymatic Fragmentation					
Human gDNA			E.coli gDNA		
Sample	Dilution	Concentration (ng/μL)	Sample	Dilution	Concentration (ng/μL)
1	1:10	11.2	1	1:4	9.53
2	1:10	8.4	2	1:4	13.2
3	1:10	7.53	3	1:4	13.1
4	1:10	10.3	4	1:4	10.0
5	1:4	16.3	5	1:4	10.5
6	1:10	13.3	6	1:4	10.3
7	1:4	22.8	Positive Control 1	1:4	21.7
8	1:4	22.5	Positive Control 2	1:4	24.5
Positive Control 1	1:10	14.8	Average Undiluted Library	-	56.4
Positive Control 2	1:10	17.1	Undiluted Std.	-	23.1
Positive Control 3	1:10	23.5			
Average Undiluted Library	-	112			
Undiluted Std.	-	50.1			

Sequencing bias: Significant GC bias resulted from the sonication parameters listed in S. Table 5. These sonication parameters over-fragmented the adenine-thymine (AT)-rich portions of DNA. This occurred because of the weaker A-T bond (two hydrogen bonds) compared to the much

stronger G-C bond (three hydrogen bonds). Size selection occurs throughout both library preparation assays to remove small and inutile DNA fragments, in this case disproportionately removing AT-rich portions of the sample genome. At the time of sample sequencing, we observed drastic pro-GC bias as seen in S. Fig. 4. The normalized coverage should ideally be a flat line at 1. We ultimately used different input DNA with more optimized sonication parameters, however these results represent a significant problem with DNA fragmentation methods and NGS library preparation in general. This problem may be addressed by lowering the sonication peak incident power and reducing the number of cycles per burst or duration of the cycle to fragment the AT-rich regions to a lesser extent.

Supplementary Figure 4: A compilation of positive control and on-deck mechanically fragmented human DNA libraries exhibiting significant pro-GC bias. Ideal normalized coverage is a flat line at 1, with variation between 1.4 and 0.4.

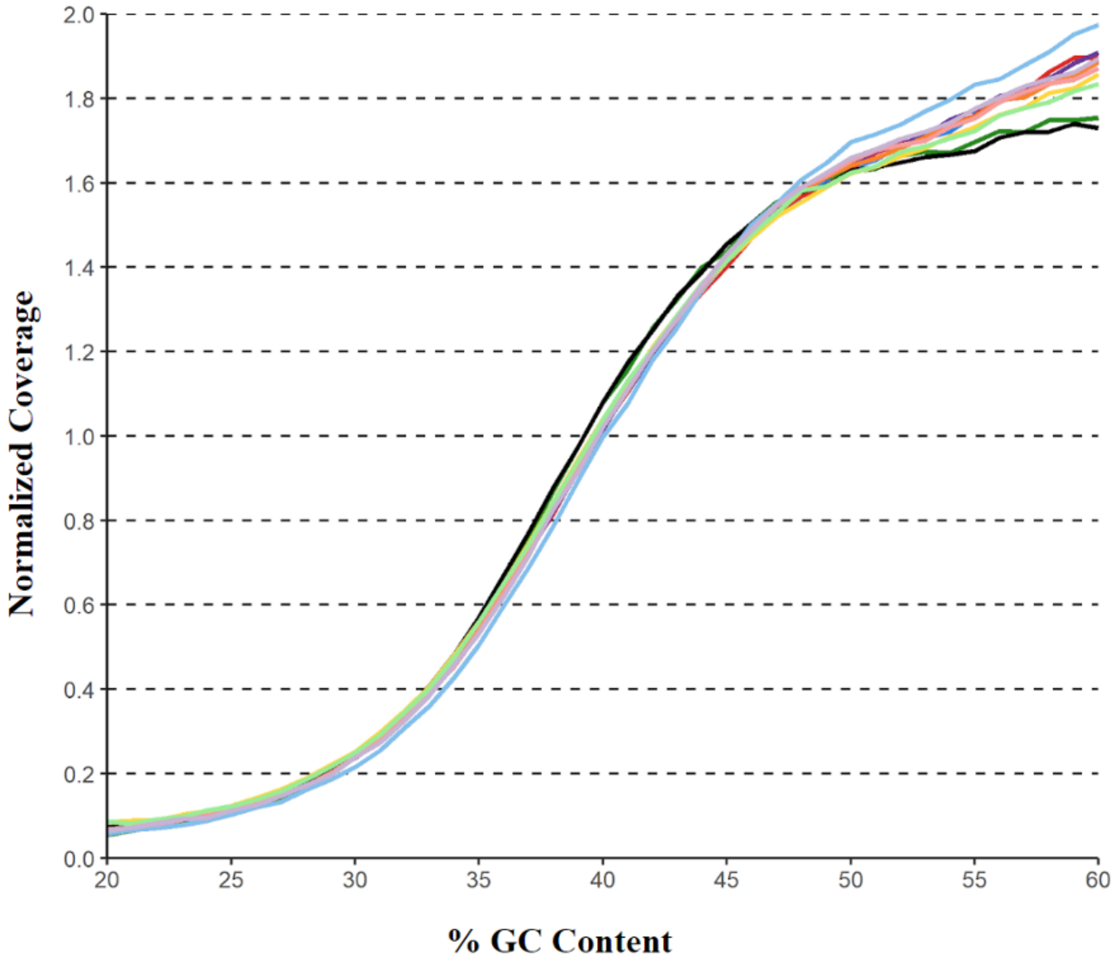

Supplementary Table 5: DNA sonication parameters that produced significant pro-GC bias. DNA was sonicated using a Covaris S220 Focused-ultrasonicator (Covaris, Woburn, MA).

**Parameter**	**Value**
Peak Incident Power	210
Duty Factor	10%
Cycles per burst	200 count
Duration	150 sec
Water level	12
Temperature	7°C

Supplementary Figure 5: Electropherograms of samples prepared on-platform without pretreatment. Note high adapter-dimer peaks (shown by arrows) at roughly 130bp, related to insufficient surface passivation.

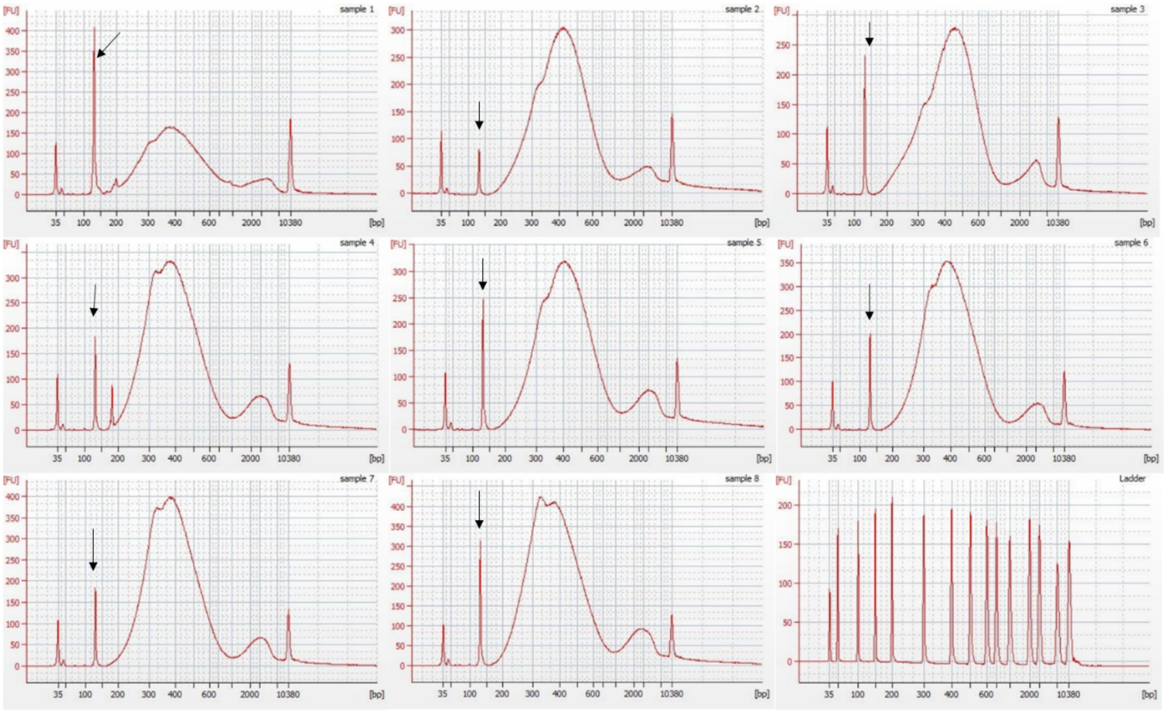

Supplement: Supplementary file 1 — Supplementary Information. [file 41598_2024_63014_MOESM1_ESM.pdf]
